# Chromatin interaction landscape of epimorphic regeneration in teleost fish

**DOI:** 10.1126/sciadv.aeh2087

**Published:** 2026-10-09

**Authors:** Xiaohui Jia, Xianglei Zhang, Weifeng Lin, Tengfei Ren, Shiqi Liu, Yufei Lou, Wei Wang

**Affiliations:** ^1^ College of Biological Sciences, China Agricultural University, Beijing 100083, China.; ^2^ National Institute of Biological Sciences, Beijing 102206, China.; ^3^ Tsinghua Institute of Multidisciplinary Biomedical Research, Tsinghua University, Beijing 100084, China.; ^4^ Graduate School of Peking Union Medical College, Chinese Academy of Medical Sciences, Beijing 100730, China.

## Abstract

Reorganization of chromatin architecture—including chromatin loops, topologically associating domains (TADs), and A/B compartments—has been increasingly linked to development and cell differentiation. However, how chromatin architecture reorganizes during the shift from homeostasis to regeneration remains poorly characterized. Here, we used zebrafish fin regeneration as a model to systematically investigate, via micro-C, the chromatin architecture associated with blastema formation. Our analyses unveiled that the alteration of chromatin looping upon amputation was dominant compared with that of TADs and compartments. Genes directly linked to injury-altered loop interactions, including essential regeneration regulators, were mainly related to morphogenesis and cell division. A side-by-side comparison between zebrafish and African killifish uncovered conserved and species-specific chromatin reorganization following injury. Notably, perturbing injury-altered loop interactions at the *il11a* locus suppressed its expression and impaired fin regeneration efficiency. Further, CTCF/CTCFL and Atf7 binding motifs were the top shared motifs enriched in regeneration-altered loop anchors. Our study uncovers the landscape of chromatin interactions during fin regeneration.

## INTRODUCTION

The eukaryotic genome is highly organized inside the nucleus to form a hierarchical three-dimensional (3D) organization named chromatin architecture (*1*). It encompasses linear gene order, basic nucleosome packaging, and higher-order structures, including chromatin loops, topologically associated domains (TADs), and chromosome compartmentalization (*2*). During development, the reorganization of chromatin architecture is important for governing gene expression linked to cell fate transitions (*3*–*6*). Alteration of chromatin structure in cells is usually accompanied with changes in DNA accessibility or the interactions between gene promoters and distal regulatory elements (*7*). Further, aberrant alterations of chromatin architecture contribute to disease progression and aging-related functional decline (*8*–*10*). Yet, how chromatin architecture reorganizes during the shift from homeostasis to regeneration remains poorly characterized. A recent study conducted in sensory neurons using Hi-C suggested that promoter-enhancer looping plays a pivotal role in axonal regeneration (*11*). To date, we still know little about many fundamental questions regarding regeneration-associated chromatin reorganization in vertebrates, including (i) what is the scale and major hierarchical type of chromatin reorganization occurring at the onset of regeneration and (ii) do regenerative species share common features of chromatin reorganization?

The caudal fin of teleost fish is an attractive model for investigating the principles of chromatin reorganization during regeneration due to its relatively simple structure, robust regenerative capacity, and well-documented cell response upon injury. A typical zebrafish caudal fin contains 16 to 18 segmented bony rays, and each ray is composed of two concave hemirays enclosing osteoblasts, fibroblasts, vascular cells, and nerves (*12*). Upon amputation, fin regeneration involves roughly five distinct stages, including wound healing, blastema formation, regenerative outgrowth, new tissue morphogenesis, and regeneration termination. Wound healing is a universal process present in both regenerative and nonregenerative species after tissue damage. In fins and limbs, blastema formation determines the onset of regeneration and prepares cells for new tissue formation (*13*, *14*). Nonetheless, the landscape of chromatin interactions involved in this critical stage is still unknown. In this study, we performed high-resolution micro-C (*15*) to compare the chromatin architecture between homeostasis and blastema formation during zebrafish and African killifish caudal fin regeneration. We found that regeneration-induced reorganization of chromatin architecture primarily occurred at the level of chromatin loops. Side-by-side comparisons indicated that regeneration-induced chromatin architecture reorganization evolved in regenerative species, leaving the presence of both species-specific and conserved features in each genome. Notably, disruption of injury-altered loop interactions at the *il11a* locus repressed the expression of *il11a* and impaired fin regeneration efficiency. Further, we found that the CTCF/CTCFL and Atf7 binding motifs were the shared top motifs enriched in the injury-altered chromatin loop anchors, implying that these transcription factors are the potential regulators controlling the dynamic chromatin looping during the early onset of regeneration. Our study uncovered the landscape of chromatin interactions during fin regeneration and shed new light on the understanding of the relation between the 3D genome organization and regeneration.

## RESULTS

### Regeneration-induced chromatin architecture reorganization largely occurred at the level of chromatin loops

After caudal fin amputation, zebrafish can complete wound healing in a few hours and initiate regeneration in 2 days (*16*). A zebrafish fin blastema forms and amplifies within 2 days postamputation (dpa), followed by regenerative outgrowth and new tissue morphogenesis (*17*, *18*). To characterize regeneration-induced genome reorganization, we applied the micro-C, a micrococcal nuclease-based Hi-C protocol, to capture the chromatin architecture in zebrafish caudal fin at 0 and 2 dpa (Fig. 1A). Our micro-C contact matrices displayed sufficient sequencing depth to discover chromatin structures including A/B compartments, TADs, and chromatin loop interactions at high resolution (Fig. 1B and fig. S1A).

**Fig. 1.**
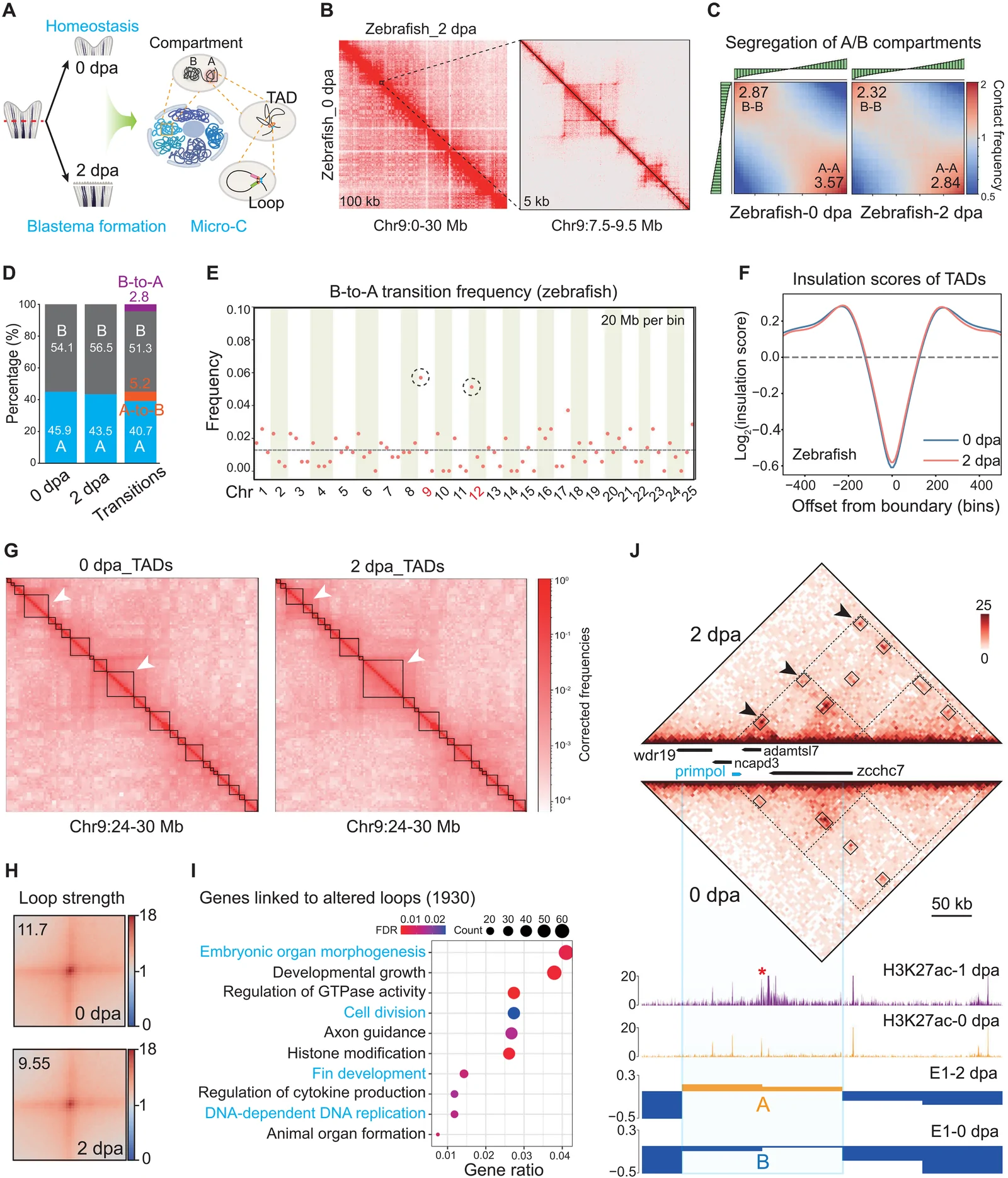
Multiscale 3D genome reorganization during zebrafish fin regeneration. (**A**) The schematic diagram showing the characterization of chromatin architecture at 0 dpa and 2 dpa during zebrafish caudal fin regeneration. Figure elements were created in BioRender. Yang, J. (2026) https://BioRender.com/bqn7p2k. (**B**) Contact map of 0 dpa (bottom left) and 2 dpa (top right) samples at 100-kb resolution in the region of Chr9:0-30 Mb. Right: a zoom-in view at 5-kb resolution. (**C**) Saddle plots of the genome-wide interaction at 0 and 2 dpa. The compartment strength was decreased upon injury. (**D**) Bar plot showing the proportion of A/B compartments at 0 and 2 dpa and the compartment transitions (B-to-A and A-to-B). (**E**) The frequency of B-to-A compartment transitions across chromosomes. Each bin represents a 20-Mb region. The dashed line represents the random frequency. (**F**) Comparison of the average insulation scores around TAD boundaries at 0 dpa and 2 dpa. (**G**) A representative location on chromosome 9 showing the TAD changes at 50-kb resolution. The white arrowheads indicated the injury-altered TADs. (**H**) Aggregated peak analysis (APA) showing the strength of detected chromatin loops at 0 dpa and 2 dpa. (**I**) Representative gene ontology (GO) terms enriched for the 1930 genes involved in injury-altered chromatin loop interactions. (**J**) Complex chromatin reorganization at the *primpol* locus upon fin amputation. Active enhancers were defined by the H3K27ac peaks, * indicates up-regulated peaks. Positive values of the eigenvector represent A compartment (orange), and negative values indicate the B compartment (blue). Arrowheads indicate enhanced chromatin loop interactions.

Chromosome compartmentalization is one of the key features of animal genomes. Although not an absolute determinant of gene expression, A compartments are generally euchromatic and transcriptionally active, while B compartments tend to be heterochromatic and transcriptionally inactive (*19*). In contrast to the acute biological change from homeostasis to regeneration, we observed a minor alteration in the segregation of A (3.57 versus 2.84, *P* < 0.01) and B (2.87 versus 2.32, *P* < 0.01) compartments, as revealed by the compartment strength scores, between 0 and 2 dpa (Fig. 1C and fig. S1B). Despite that only a small portion of the compartments exhibited A-to-B (5.2%) or B-to-A (2.8%) transition upon injury (Fig. 1D), we found a relatively enhanced frequency of B-to-A and A-to-B compartment transitions on chromosome 9, 12, and 4 compared with other chromosomes in zebrafish (Fig. 1E and fig. S1, A and C). Next, we sought to determine at what levels the major chromatin architecture changes have occurred. The analyses of TAD dynamics at 50-kb resolution showed that the insulation scores of TAD boundaries were slightly increased at the whole genome level after fin amputation (Fig. 1F), indicating that injury mildly weakened the TAD boundaries. In addition, less than 10% TAD boundaries were altered compared with the intact tissues (fig. S1D). For instance, we observed injury-induced changes of TAD domains in the regions of 24th to 30th megabases on chromosome 9 (arrowhead, Fig. 1G). These data implied that injury did not induce marked genome-wide changes in chromosome compartmentalization and TADs at the onset of fin regeneration.

Chromatin loops are defined as smaller-scale structures within TADs and function as a critical mechanism for creating physical proximity between distal regulatory elements and their target genes to control expression (*7*). To assess the global strength of identified chromatin loops, we performed aggregate peak analyses (APA). Compared with 0 dpa (11.7), the reduced APA score at 2 dpa (9.55) indicated that the overall loop interaction strength exhibited a decrease (*P* = 6.55 × 10^−11^) following injury (Fig. 1H and fig. S1E). Specifically, we identified 1518 enhanced (11.6%) and 1261 reduced (9.6%) loop interactions between 0 and 2 dpa (fig. S1F and table S1), with 1930 genes (average TPM > 1) directly linked to the loop anchors. Among these anchor genes, 18% were significantly up-regulated, while 11% were down-regulated following injury (fig. S1G). Gene ontology (GO) analysis suggested that the representative enriched GO terms were related to embryonic organ morphogenesis, cell division, fin development, and DNA-dependent DNA replication (Fig. 1I and table S1). Cell division is crucial for activating regeneration in different tissues and organs. We detected sophisticated reorganization of chromatin architecture at the primase and DNA directed polymerase (*primpol*) locus and the downstream regions (Fig. 1J). Primpol is a primase that functions in both nucleus and mitochondria, which allows the initiation of DNA synthesis de novo using deoxynucleotides (*20*, *21*). We observed both B-to-A compartment transition and enhanced long-range chromatin loop interactions (arrowheads) in this region at 2 dpa (Fig. 1J). Moreover, we also detected boosted interactions at the *mki67* locus, a classic marker of cell proliferation, between its promoter and adjacent gene promoters (fig. S1H). This observation can be further validated by the chromosome conformation capture (3C) assays (fig. S1I). Our results suggested that the alteration of chromatin loop interactions in response to injury was relatively more robust compared with TADs and compartments.

### Genes essential for regeneration are involved in injury-induced chromatin loop dynamics

Successful initiation of regeneration requires the activation of regeneration-responsive genes (RRGs) directing blastema formation and proliferation. To understand the association between chromatin architecture reorganization and the expression of key RRGs, we performed an integrated analysis using the micro-C data, bulk RNA sequencing (RNA-seq) data, and the publicly available single-cell RNA-seq data collected at 0 and 2 dpa during zebrafish fin regeneration (fig. S2A) (*22*). Our analysis identified 45 blastema marker genes directly linked to the injury-altered loop interactions (Fig. 2A and table S2), including the classic blastema markers *aldh1a2* and *msx1b* (Fig. 2B and fig. S2B) (*23*, *24*). *Aldh1a2*, encoding a rate-limiting enzyme that catalyzes the conversion of retinaldehyde into retinoic acid (RA), is a conserved regeneration regulator involved in distinct organs and species (*25*–*27*). It has been shown that RA signaling activities play essential roles in blastema formation, proliferation, and survival during zebrafish fin regeneration (*28*). The micro-C detected enhanced long-range loop interactions between the *aldh1a2* promoter and downstream enhancers at 2 dpa (Fig. 2C). Taking one of the observed chromatin contacts as an example, we successfully validated the interaction using 3C assays (Fig. 2D and fig. S2C), indicating a relatively high quality of the data. Unlike *aldh1a2*, reduced loop interactions were detected between the *msx1b* promoter and distal regulatory elements after fin amputation (fig. S2D).

**Fig. 2.**
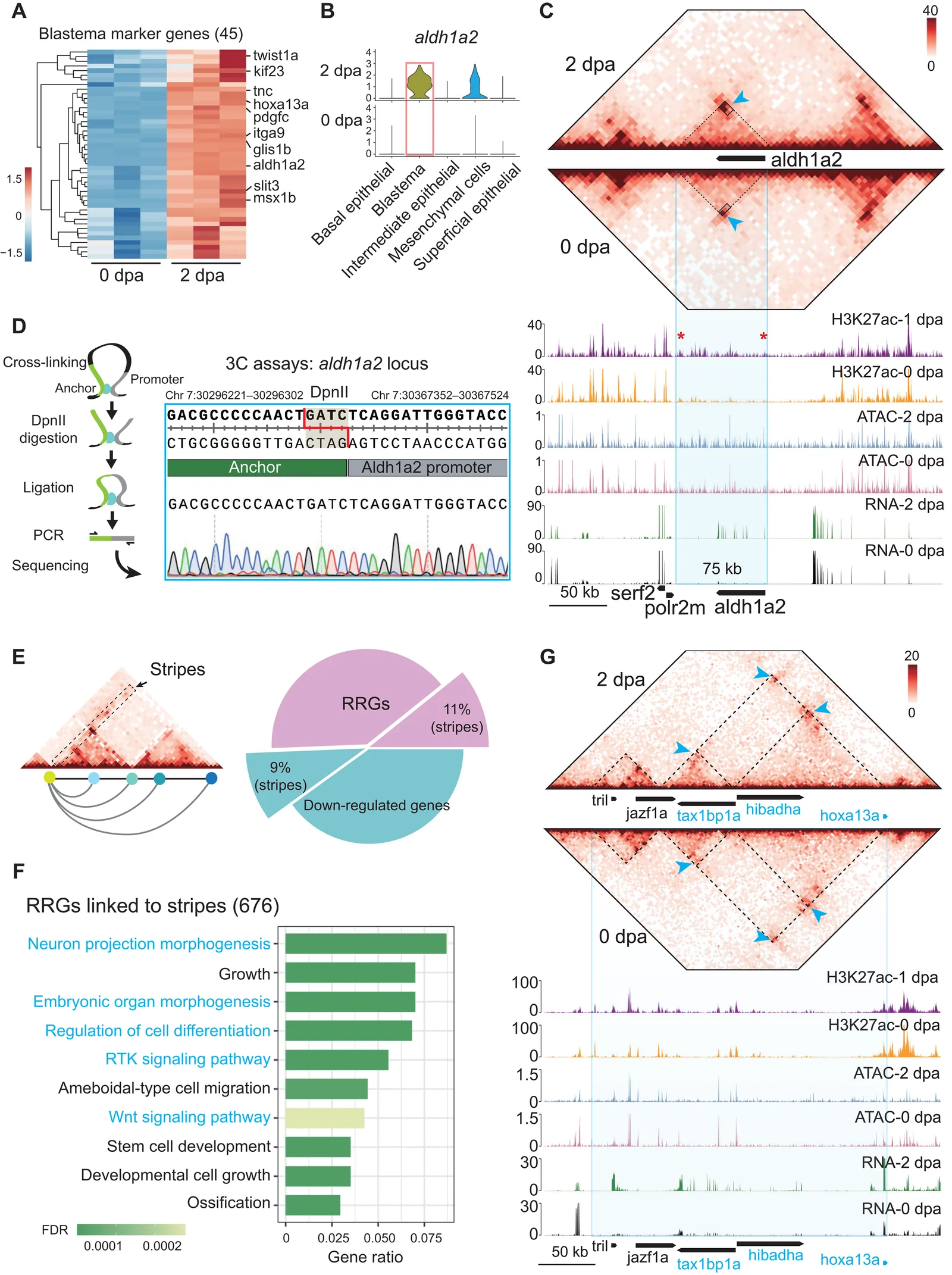
Injury-induced chromatin loop dynamics is associated with RRGs involved in blastema formation. (**A**) Heatmap of 45 blastema marker genes linked to the injury-altered loop interactions. (**B**) Violin plot showing the expression of *aldh1a2* during zebrafish fin regeneration. *Aldh1a2* expression is mainly enriched in the blastema. (**C**) An enhanced promoter-enhancer interaction (arrowhead) was detected at the *aldh1a2* locus during zebrafish fin regeneration. Gene expression and active enhancers were visualized through the RNA-seq peaks and H3K27ac peaks, respectively. * indicates up-regulated peaks. (**D**) Validation of the promoter-enhancer interaction at the *aldh1a2* locus [arrowhead in (C)] using 3C assays. Left: the schematic representation of the 3C workflow. Right: confirmation of the PCR product derived from 3C assays via Sanger sequencing. (**E**) The percentage of RRGs linked to the architectural stripes. Left: a diagram showing the definition of a stripe, in which a genomic region robustly contacts with many adjacent regions. (**F**) Representative GO terms enriched for the 676 RRGs linked to stripes during zebrafish fin regeneration. (**G**) Architectural stripe formed at the *hoxa13a* locus. Three RRGs were involved in this stripe including *hoxa13a*, *tax1bp1a*, and *hibadha*. Arrowheads indicate the enhanced chromatin interactions at 2 dpa compared with 0 dpa.

Previous studies have shown that architectural stripes usually function as hubs for gene regulation by looping together multiple active enhancers and promoters over long genomic distances, regulating cell identity genes critical for development and disease (*29*, *30*). Such stripes are linear structures formed within TADs in Hi-C/micro-C contact maps (*31*), in which a genomic region robustly contacts with many adjacent regions (Fig. 2E). Using the tool StripeCaller, 1863 stripes at 0 dpa and 2193 stripes at 2 dpa were identified in zebrafish (table S3). As expected, we detected the formation of stripes when a promoter interacted with multiple enhancers or an enhancer interacted with multiple other enhancers (fig. S2, E and F). For example, the promoter (green) of gene *sp9*, encoding a transcription factor involved in the formation of regeneration epithelium (*32*), interacted with H3K27ac-defined active enhancers (fig. S2E). Formation or disruption of stripes upon injury was detected for regulators of key regeneration-related signaling pathways, including *lzts2a* (Wnt signaling) and *spry2* (Fgf signaling), respectively (fig. S2, G and H).

To further elucidate the contribution of stripes in the regulation of regeneration, we examined 3178 RRGs (log_2_FC > 0.75, FDR < 0.05, logCPM > 1; table S2) activated at 2 dpa and found that ∼11% (676) of them were linked to the architectural stripes (Fig. 2E). The representative top GO terms enriched for the stripe linked RRGs were neuron projection morphogenesis, embryonic organ morphogenesis, regulation of cell differentiation, and RTK and Wnt signaling pathways (Fig. 2F and table S3), which are essential biological processes involved in regeneration. For example, Hox genes have been shown to play critical roles in controlling positional information and developmental program during appendage regeneration (*33*–*35*). In zebrafish caudal fin, *hoxa13a* and neighboring RRGs (*tax1bp1* and *hibadha*) form an architectural stripe to modulate multigene expression upon injury (Fig. 2G). Our findings suggested that injury-induced dynamic loop interactions were linked to the expression of genes essential for distinct regenerative processes.

### Injury triggers increased distance of the chromatin loop interactions

Chromatin looping is a major mechanism controlling interactions among regulatory elements (promoters, enhancers, and others) in both long and short ranges. Regeneration-responsive enhancers (RREs) are critical for regeneration-dependent gene activation (*18*, *36*). It has been reported that the shared RRE-regulated genes identified during zebrafish and African killifish fin regeneration are related to embryonic morphogenesis, regulation of cell migration, and angiogenesis (*18*), while the species-specific ones exhibited divergent enrichment in signaling pathways (fig. S3, A to C). Since a given enhancer can regulate the expression of a distant gene or multiple neighboring genes, the precise assignment of target genes to H3K27ac ChIP-seq– or ATAC-seq–identified enhancers remains challenging in the field. Our micro-C data bridged the gap by directly capturing the enhancer-promoter interactions. For example, our data not only validated the interaction between a previously characterized RRE *Z-IEN* and its corresponding target *inhbaa* (*18*) but also discovered previously unidentified nested chromatin interactions at this locus including the *Z-IEN* involved enhancer-enhancer interaction (arrow) and the interaction between *inhbaa* promoter and a new RRE *Z-IEN2* (fig. S3D). Transgenic reporter assays showed that *Z-IEN2* was sufficient to drive green fluorescent protein (GFP) expression in an injury-dependent manner during fin regeneration (fig. S3E). Further, long-range interactions between RREs and distant gene promoters, or interactions that skip neighboring genes, can be identified in our dataset (fig. S4, A to C). By leveraging published zebrafish tail fin single-cell ATAC-seq data (*37*), we were able to resolve the cell type specificity (e.g., blastema and epidermis) of the identified RREs and their associated loop interactions (fig. S4, D and E). The motif enrichment analysis of these cell type–specific RREs uncovered differential deployment of transcription factor binding motifs between blastema (e.g., Tcf4 and Hoxd13) and epidermis (Klf4 and Klf5) in addition to the shared AP-1 motifs (fig. S4, F and G).

Previously, many studies highlighted the significance of promoter-enhancer interactions during cell fate specification, embryogenesis, and diseases (*6*, *38*, *39*). To understand whether certain dominant types of loop interactions were present during regeneration, we compared the proportion of loop types between 0 and 2 dpa. Our results showed that injury did not trigger substantial alterations in the proportion of promoter-enhancer, promoter-promoter, enhancer-enhancer, and other types (e.g., nonenhancer- or nonpromoter-related interactions) (Fig. 3A). Unexpectedly, the promoter-enhancer interactions only occupied a small portion of chromatin interactions detected in the genome, with “other types” being dominant (Fig. 3A). Combined with previously identified silencer elements during zebrafish fin regeneration (*40*), we found the unclassified interaction types included, at minimum, physical contacts between silencers and promoters or enhancers (fig. S5A). However, among the altered chromatin loops following amputation, we detected relatively more robust changes: a decrease in the proportion of promoter-enhancer and enhancer-enhancer interactions and an increase in that of other types (Fig. 3A). Furthermore, we investigated the preference of loop distance upon injury. A comparison of loop length between 0 and 2 dpa showed that regeneration induced a significant (*P* < 0.001) increase in the distance of loop interactions (Fig. 3B). Consistent trend was also detected when we only focused on the loops involving gene promoters or injury-altered loops (*P* < 0.05; Fig. 3B). Taking the *itga9* locus as an example, we identified dynamic switches in loop interactions at different distances, characterized by the reduction of a shorter-range (132 kb) interaction and the enhancement of a longer-range (194 kb) interaction (Fig. 3C).

**Fig. 3.**
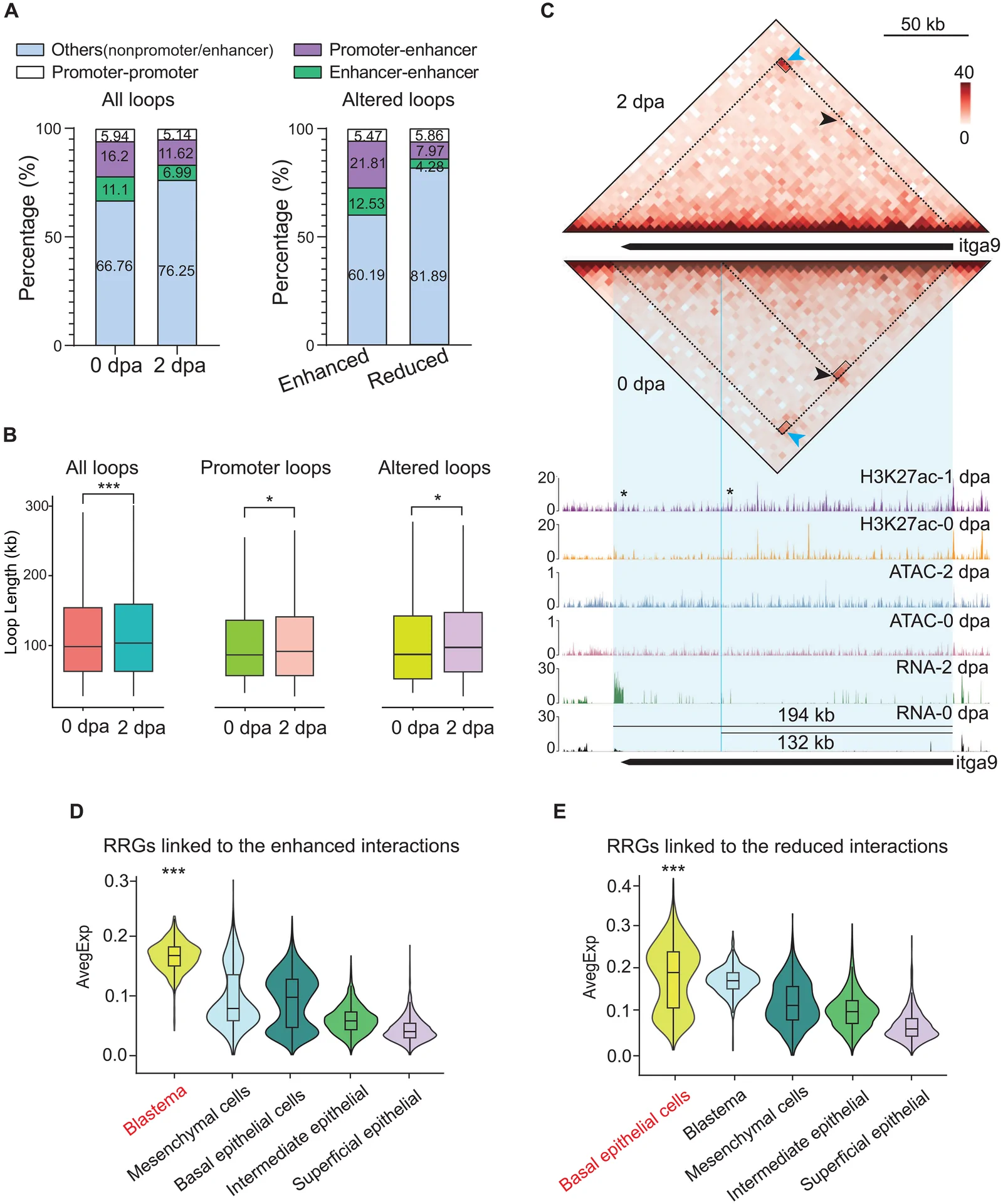
Injury increases the distance of chromatin loop interactions in zebrafish. (**A**) Stacked bar plots showing the proportion of different types of chromatin loop interactions including promoter-promoter, promoter-enhancer, enhancer-enhancer, and others. The type others refers to interactions involving nonpromoter/enhancer-related regulatory elements. All loops (left) detected at 0 and 2 dpa and injury-altered loops (right) were examined. (**B**) The distance of chromatin loop interactions was increased at 2 dpa compared with 0 dpa. Loop lengths were evaluated for all loops, promoter-related loops, and injury-altered loops. ***P < 0.001 and *P < 0.05 (Wilcoxon tests). (**C**) Detection of both enhanced (blue arrowhead) and reduced (black arrowhead) chromatin loop interactions at the *itga9* locus after fin amputation. Gene expression and active enhancers were visualized by the RNA-seq peaks and H3K27ac peaks, respectively. *up-regulated peaks. (**D**) The expression of RRGs linked to the enhanced chromatin loop interactions was mainly enriched in blastema cells. ***P < 0.001 (Wilcoxon tests). (**E**) The expression of RRGs linked to the reduced chromatin loop interactions was mainly enriched in the basal epithelial cells. ***P < 0.001 (Wilcoxon tests).

Next, we explored what cell populations were associated with injury-induced loop dynamics. We analyzed the enrichment of gene expression in different cell populations for 458 RRGs directly linked to the injury-altered loop anchors (table S1). We found that RRGs linked to the enhanced and reduced chromatin loops were mainly enriched in blastema cells (*P* < 0.001; Fig. 3D) and basal epithelial cells (*P* < 0.001; Fig. 3E), respectively. Many of these RRGs with known regeneration-related functions, including *flrt3*, *ccn4a*, *fras1*, *dachc*, and *glis1b*, displayed long-range interactions at 2 dpa (fig. S5, B to F). For instance, we detected an injury-induced increase in long-range chromatin interactions at 2 dpa surrounding *glis1b*, a gene that acts as a vital inducer of pluripotency (*41*). This interaction linked the *glis1b* promoter to a 155-kb downstream genomic region marked by elevated H3K27ac signal peaks (fig. S5F). Collectively, our findings suggested that regeneration triggered a significant increase in the distance of chromatin loops and underscored an underappreciated involvement of nonpromoter/enhancer-associated interactions during blastema formation.

### CTCF binding motifs are highly enriched in injury-altered loop anchors in zebrafish and African killifish

Regeneration occurs in a wide range of animal lineages (Fig. 4A). To investigate whether our observations in zebrafish were evolutionarily conserved in other regenerative species, we explored the chromatin architecture during African killifish fin regeneration. Combined with the synteny analysis between the two species, our results showed that ∼60% of one-to-one conserved genes were involved in the same type of compartment (43.3% in A and 17.2% in B compartment; fig. S6A). Like zebrafish, a minor change was detected for the segregation of A/B compartments, the insulation scores of TAD boundaries, and chromatin loop types (Fig. 4B and fig. S6, B to D). At the genome level, we also observed a significant reduction in loop interaction strength (17.9 versus 14.3, *P* = 2.74 × 10^−7^) during killifish regeneration (Fig. 4C and fig. S6E). Despite less than 5% of compartments showing A-to-B or B-to-A transitions, killifish exhibited a robust enrichment of B-to-A compartment transitions on chromosome 4 (Fig. 4D and fig. S6F), whereas the homologous regions in zebrafish did not show similar enrichment (fig. S6, G and H). To understand whether there was a functional link between such large-scale compartmental reorganization and injury-dependent gene regulation, we examined the expression of RRGs located in this region at distinct stages of fin regeneration using the available African killifish bulk RNA-seq data (*18*). The highest number of RRGs was detected at 3 dpa but not 2 dpa (Fig. 4E). Among these RRGs, *disp1*, *bub1*, *bmp2b*, *esco2*, *runx2*, *dll4*, and *ccnk* were known regulators of tissue regeneration. This result supported a previous observation that switching a region from the B to A compartment does not lead to immediate gene activation but establishes a permissive chromatin environment for future regulation (*42*).

**Fig. 4.**
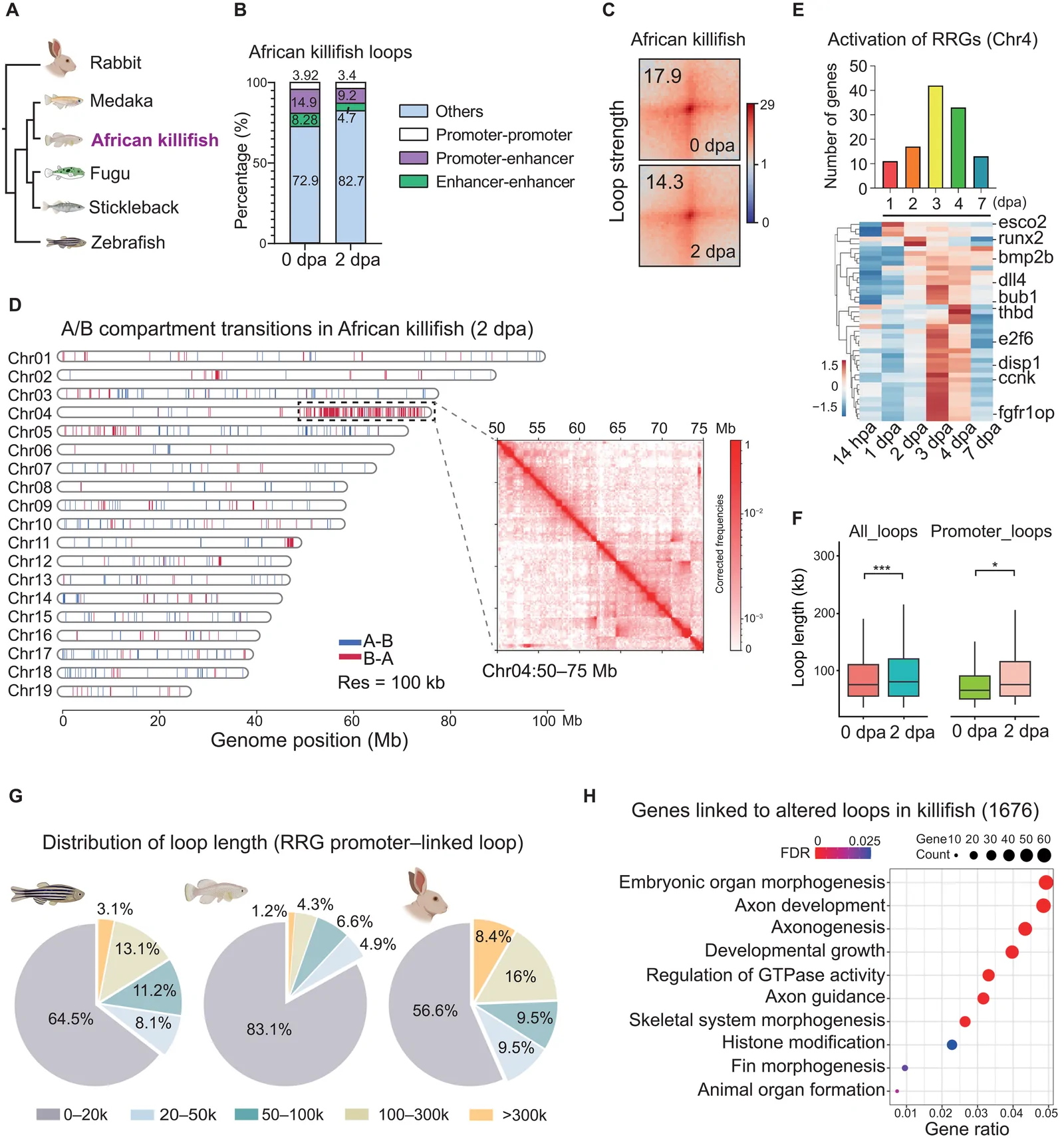
Conservation and evolution of chromatin architecture in regenerative species. (**A**) A phylogenetic tree showing the evolutionary relationships among the regenerative species including zebrafish and African killifish. Species icons were created in BioRender. Yang, J. (2026) https://BioRender.com/bqn7p2k. (**B**) Stacked bar plots showing the proportion of different types of chromatin loop interactions detected at 0 and 2 dpa during African killifish fin regeneration. (**C**) Aggregated peak analysis showing the decrease of global loop strength after injury in African killifish. (**D**) A robust enrichment of B-to-A compartment transitions was detected on chromosome 4 at 2 dpa in African killifish. Red, B-to-A transition; blue, A-to-B transition. All transitions were plotted on each chromosome. Right: the contact heatmap of the chromosome region with enriched B-to-A transitions. (**E**) Number of activated RRGs located in the genomic region with enriched B-to-A transitions on chromosome 4 (D) during different time points of African killifish fin regeneration. Bottom: heatmap showing the expression of the activated RRGs (top graph) during fin regeneration. (**F**) The distance of chromatin loop interactions was increased at 2 dpa compared with 0 dpa in African killifish. Loop lengths were evaluated for all loops and promoter-related loops. ***P < 0.001 and *P < 0.05 (Wilcoxon tests). (**G**) Distribution of interaction distance for the RRG promoter-linked chromatin loops in regenerative species, including zebrafish, African killifish, and rabbits. (**H**) Representative GO terms enriched for the 1676 genes involved in injury-altered chromatin loop interactions in African killifish.

Since the distance of loop interactions was increased during zebrafish fin regeneration, we compared the loop lengths under distinct conditions. The analyses showed that the interaction distances of all detected loops (*P* < 0.001) or the promoter-linked loops (*P* < 0.05) were also significantly longer at 2 dpa compared with 0 dpa in African killifish (Fig. 4F). Further, we examined the percentage of long-range interactions (>50 kb) for RRGs detected in fin regeneration (zebrafish and killifish) and rabbit ear pinna regeneration. Notably, long-range interactions exhibited relatively large variations among the three species in descending order as rabbit (33.9%), zebrafish (27.4%), and killifish (12.1%) (Fig. 4G).

Next, we analyzed the altered loop interactions in African killifish and identified 1131 enhanced and 440 reduced loops (table S4). Among these anchor genes, 9% were significantly up-regulated, and 11% were down-regulated following injury (fig. S6I). Genes linked to these altered loops with detectable expression were enriched in similar biological processes observed in zebrafish, including embryonic organ morphogenesis, axon development, fin morphogenesis (Figs. 1I and 4H). To investigate the functional significance of chromatin interactions in response to injury, we focused on enhancer-promoter interactions detected at the genomic loci of *fn1* and *il11a*, two conserved genes activated during the regeneration of distinct organs (*43*, *44*). Specifically, the enhancers, *RRE^fn1^*and *RRE^il11a^*, formed distinct chromatin loops with the promoters of *fn1* and *umps* (Fig. 5A) and the *il11a* promoter (Fig. 5B and fig. S6J), respectively. Transgenic reporter assays showed that *RRE^fn1^* was sufficient to drive GFP expression at the wounding site upon amputation (Fig. 5C), indicating the robustness of our approach in identifying enhancers. Further, we carried out targeted deletion of the enhancers *RRE^fn1^* and *RRE^il11a^* to disrupt chromatin interactions and examined the expression of associated targets. The results showed that deletion of *RRE^fn1^* did not affect the expression of *fn1* and *umps* (Fig. 5D), which was likely due to the presence of redundant enhancers (Fig. 5A, rectangle). In contrast, *RRE^il11a^* deletion diminished the expression of *il11a* and significantly impaired fin regeneration efficiency in African killifish (Fig. 5, D to G). Single-cell RNA-seq data (*18*) showed that *il11a* was activated in the blastema and basal epidermal cells upon amputation (fig. S6J). Our data highlighted that injury-altered chromatin loop interactions were critical for the transcriptional output of target genes and efficient regeneration.

**Fig. 5.**
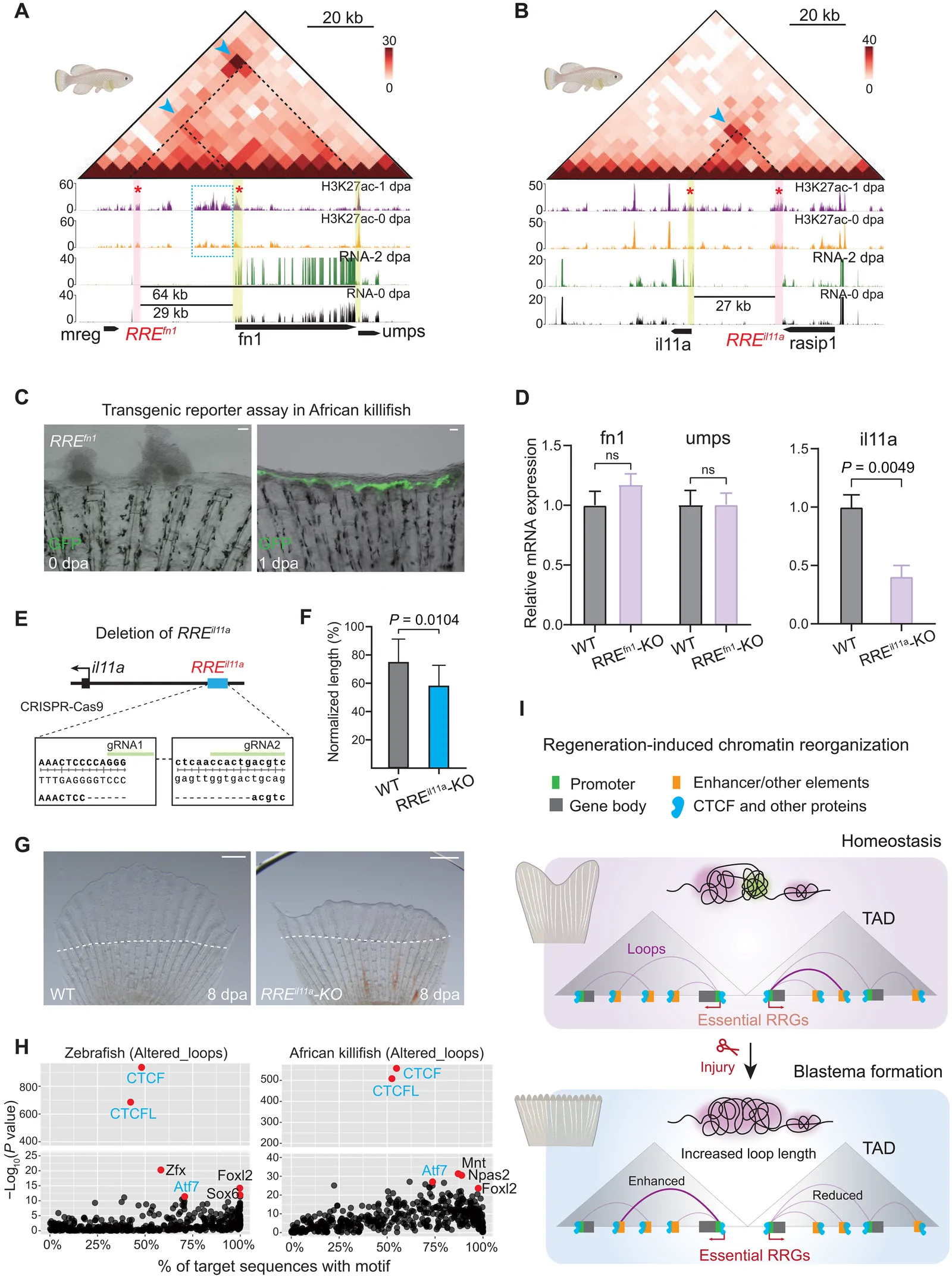
Enrichment of CTCF and ATF binding motifs in regeneration-altered loop anchors. (**A**) Promoter-RRE interactions (arrowhead) were detected at the *fn1* and *umps* locus during killifish fin regeneration. Gene expression and active enhancers were visualized through the RNA-seq peaks and H3K27ac peaks, respectively. *up-regulated peaks. (**B**) A promoter-RRE interaction (arrowhead) was detected at the *il11a* locus during killifish fin regeneration. Gene expression and active enhancers were visualized through the RNA-seq peaks and H3K27ac peaks, respectively. *up-regulated peaks. (**C**) *K-RRE^fn1^* was sufficient to drive GFP expression at 1 dpa during African killifish fin regeneration. Scale bar, 100 μm. (**D**) Left: the relative mRNA expression of *fn1* and *umps* in the *RRE^fn1^*-KO mutants (*n* = 40). Right: the relative mRNA expression of *il11a* in the *RRE^il11a^*-KO mutants (*n* = 20). (**E**) Generation of *RRE^il11a^*-KO mutants. Top; schematic diagram showing the deletion of *RRE^il11a^* through CRISPR-Cas9. Bottom: sequencing confirmation of *RRE^il11a^*-KO mutants. (**F**) Qualification of fin regeneration between wild-type (WT) and *RRE^il11a^*-KO animals at 8 dpa, *n* = 13. (**G**) Representative caudal fin images of WT and *RRE^il11a^*-KO at 8 dpa. Scale bars, 500 μm. (**H**) Motifs enriched in regeneration-altered loop anchors in zebrafish (left) and African killifish (right). (**I**) A model elucidating the injury-induced chromatin architecture reorganization during caudal fin regeneration. ns, not significant.

Ample evidence has shown that the CTCF-cohesin complex is a master regulator of chromatin looping during development and disease (*45*–*47*). Therefore, we asked whether regeneration deployed the same or a distinct regulator by performing motif enrichment analyses with injury-altered loop anchors. CTCF and CTCFL were the most enriched transcription factor binding motifs in both zebrafish and African killifish (Fig. 5H). Since the cohesin complex does not have a sequence-specific DNA binding motif, we asked whether the expression of its subunits was induced after injury. Our analysis showed that certain cohesin subunits like *smc1al*, *smc3*, *rad21a*, *stag2b*, *nipblb*, *waplb*, and *pds5a* were highly enriched in blastema compared with other cell types (fig. S6K), indicating the involvement of cohesin complex during regeneration. Besides, the DNA sequences recognized by the Activating transcription factor 7 (Atf7) were another shared motif with significant enrichment and detectable caudal fin expression. Atf7 functions as a stress-responsive and DNA binding factor that can form heterodimers with the AP-1 complex, a key regulator directing regeneration and activation of RREs (*48*, *49*). Together, our data indicate that injury-induced remodeling of chromatin architecture harbors both conserved and species-specific signatures in highly regenerative teleost fish, which together orchestrate efficient regeneration. CTCF-cohesin complex and other transcription factors like Atf7 are the potential regulators controlling the dynamic chromatin looping at the onset of regeneration.

## DISCUSSION

Why regeneration only occurs in some animal lineages but not others is still a puzzle in biology. Genome evolution is one of the critical contributors driving the diversity in animal morphology, behavior, and physiology, particularly among distantly related species (*50*–*52*). Increasing evidence suggests that both similarities and differences are present in 3D genome organization across species, and evolution seems to tolerate substantial changes in such organization (*53*). In this study, we generated high-quality micro-C datasets in zebrafish and African killifish caudal fins to understand the chromatin architecture associated with epimorphic regeneration. We showed that alterations in chromatin looping were the primary changes induced after amputation. The relatively mild changes in chromosome compartmentalization and TADs imply that large-scale global genome reorganization was not required for regeneration. This is consistent with the established view that chromatin looping is highly dynamic and permits rapid, reversible reorganization within the nucleus.

Following fin amputation, certain distant genomic regions (e.g., enhancers, promoters, and other regulatory elements) were brought into close physical proximity to form context-dependent loop interactions (Fig. 5I). Diverse chromatin loops were continuously formed, broken, and remodeled by CTCF and other factors through loop extrusion. Consequently, enhancers, promoters, and other regulatory elements could establish context-dependent interactions. During this process, many long-range chromatin interactions were engaged in genomic loci essential for regeneration (e.g., *aldh1a2*, *primpol*, *bub1*, *itga9*, *dachc*, and *runx2*), highlighting the potential significance of regulatory inputs from distal elements that were silenced under homeostatic conditions.

It has been widely recognized that many conserved RRGs and signaling pathways (e.g., Wnt, BMP, FGF, and retinoic acid) function as common regulators controlling regeneration from lower animals, such as hydra, planarians, and zebrafish, to mammals like mice and rabbits (*25*, *54*, *55*). Differential regulation of these key regulators, mediated by evolutionary changes in enhancers, between regenerative and nonregenerative species, has been proposed as an important mechanism for the diversity of regenerative capacity (*18*, *25*). Evolutionary modulation of chromatin architecture could be a potential source for creating gene regulatory changes in RRGs. This argument was supported by our observation that African killifish have species-specific enrichment of the B-to-A compartment transitions on chromosome 4. When such alterations arise in a genomic region harboring essential RRGs, the regenerative capacity of the species may become impaired. Together, our work establishes the chromatin interaction landscape of epimorphic regeneration and provides new insights for understanding whether chromatin architecture reorganization contributes to regeneration.

## MATERIALS AND METHODS

### Fish husbandry

All the zebrafish experiments were done using the AB wild-type line at 3 months of age. The inbred *Nothobranchius fuzeri* GRZ strain at 2 months of age was used for all the African killifish–related experiments. The water temperature was maintained at 28°C for zebrafish and 26°C for African killifish. All animal procedures in this study were approved by the NIBS Laboratory Animal Management Committee under the license number NIBS2022M0006, and all experimental processes followed the relevant ethical regulations. All animal experiments in this study were repeated at least twice.

### Fish caudal fin amputation, collection, and cell dissociation

All fish were anesthetized with MS-222 (150 mg/liter), followed by fin amputation (half of the tail). For the micro-C experiments, two replicates were prepared for 0 dpa and 2 dpa. Specifically, 16 zebrafish and 10 African killifish were used to collect the intact tissues (0 dpa) and regenerating blastema (2 dpa) for each replicate. For cell dissociation, the collected tissues were pooled, cut into small pieces, and then digested with collagenase type II (200 U/ml; Worthington Biochemical, LS004174) in DPBS at 28°C for 20 min. The digestion was neutralized with an equal volume of 5% fetal bovine serum (FBS) in DPBS, and the supernatant containing dissociated cells was centrifuged, resuspended with 5% FBS in DPBS, and chilled on ice. The undigested tissues were redigested with collagenase type II (200 U/ml) in DPBS at 28°C for an additional 20 min and neutralized with 5% FBS in DPBS. The dissociated cells were also centrifuged, resuspended in a new tube containing 5% FBS in DPBS, and chilled on ice. To obtain enough cells, the redigestion processes were repeated twice. The suspensions obtained from three digestions were mixed and filtered through a 100-μm cell strainer to remove undigested tissue pieces, and the filtered cell solution was centrifuged at 500*g* for 5 min at 4°C. With resuspension in 5% FBS in DPBS, the cell viability was detected using trypan blue (Proteintech, PR40016) to ensure 90% or higher survival rate before moving into the next step.

### Micro-C library preparation

Previously published micro-C procedures were followed with modifications (*15*, *25*). The dissociated cells were first cross-linked with 1% formaldehyde (Thermo Fisher Scientific, 28906) for 10 min at room temperature (RT). The reaction was quenched with 2 M tris buffer (pH 7.5) for 5 min, followed by washing twice with DPBS. Subsequently, the cells were subjected to a second cross-linking step using 3 mM DSG (Thermo Fisher Scientific, 20593) for 45 min, after which the reaction was quenched with tris buffer for 5 min and followed by two additional washing steps with DPBS. Then, the fixed cells were aliquoted into 0.5 to 1 million cells per tube. Afterward, the cross-linked cells were incubated on ice with 100 μl of complete MB #1 buffer [50 mM NaCl, 10 mM tris-HCl (pH 7.5), 5 mM MgCl_2_, 1 mM CaCl_2_, 0.2% NP-40, and 1× EDTA-free protease inhibitor cocktail (PIC; Roche)] for 20 min. After centrifugation at 10,000*g* for 5 min, the cell pellet was washed and resuspended in complete MB #1 buffer. Chromatin was then fragmented by incubating with MNase (Worthington Biochemical, LS004798) at an optimized concentration for 10 min at 37°C in a thermomixer C (Eppendorf) with shaking at 850 rpm. This treatment was designed to yield a fragment distribution enriched in mononucleosomes (∼80%). The digestion was terminated by adding 0.5 M EGTA solution (final concentration: 4 mM) and incubating at 65°C for 10 min.

After fragmentation, the cell pellet was washed twice with ice-cold MB#2 buffer and centrifuged. To enhance the efficiency of T4 DNA ligation, the cell pellet was incubated with 50 μl of premix solution [5 μl of 10× NEBuffer 2.1, 1 μl of 100 mM adenosine triphosphate (ATP), 2.5 μl of 100 mM dithiothreitol, 34 μl of H_2_O, and 2.5 μl of T4 PNK (10 U/μl)] for 15 min at 37°C. Then, large Klenow fragment (NEB, M0210L) was added with incubation for another 15 min. After that, biotinylated nucleotides were incorporated by incubating the sample with 25 μl of premix buffer [5 μl of 1 mM biotin-dATP, 5 μl of 1 mM biotin–2′-deoxycytidine 5′-triphosphate, 0.5 μl of 10 mM dTTP + 2′-deoxyguanosine 5′-triphosphate, 2.5 μl of 10× T4 DNA ligase buffer, 0.125 μl of bovine serum albumin (BSA; 20 mg/ml), and 11.875 μl of ddH_2_O] for 45 min at RT. Then, the reaction was quenched using 500 mM EDTA to a final concentration of 30 mM, followed by incubation at 65°C for 20 min. After that, the chromatin was centrifuged and washed with ice-cold MB#3 buffer.

Subsequently, proximity ligation was performed, and the chromatin pellet was resuspended in 250 μl of T4 reaction solution, which comprised 25 μl of 10× T4 DNA ligase buffer with ATP, 1.25 μl of BSA (20 mg/ml), and 12.5 μl of T4 DNA ligase (400 U/μl; NEB, M0202). The suspension was then incubated for over 2.5 hours at RT with slow rotation. Following centrifugation (16,000*g*, 5 min, 4°C), the pellet was treated with 100 μl of exonuclease reaction solution, containing 10 μl of 10× NEBuffer #1, 5 μl of exonuclease III (100 U/μl), and 85 μl of ddH_2_O, for a 15-min incubation at 37°C with interval mixing. To reverse cross-linking and degrade proteins, the chromatin solution was incubated overnight at 65°C with proteinase K, 10% SDS, and ribonuclease A (RNase A). Last, the DNA was purified through phenol:chloroform:isoamyl alcohol extraction and ethanol precipitation. The target DNA fragments, ranging from 250 to 400 bp, were isolated by excising the corresponding bands from a 1.5% agarose gel and were purified using a Zymoclean Gel DNA Recovery Kit (ZymoResearch, D4001) for library construction.

Following incubation of the purified DNA ligation products with Dynabeads MyOne Streptavidin C1 beads (Invitrogen, 65001), the subsequent steps of end repair, adapter ligation, and library amplification were performed using the VAHTS Universal Pro DNA Library Prep Kit for Illumina (Vazyme, ND608-02). The 150-bp paired-end sequencing was performed for all libraries.

### Micro-C data processing

The zebrafish and African killifish reference genomes and corresponding annotation files were downloaded from the Ensembl database (version 104). The rabbit micro-C data were downloaded from a recently published study (*25*) and realigned to the latest reference rabbit genome mOryCun1.1 downloaded from National Center for Biotechnology Information (NCBI).

BWA-MEM (version 0.7.17-r1188) software was used to map micro-C paired-end reads to reference genomes. The ligation junctions (Hi-C pairs) were detected and saved as “.pair” files with Pairtools (version 0.3.0) software. Pairtools was also used to sort and remove polymerase chain reaction (PCR) duplicates in the .pair files. To generate contact maps with higher resolution, two replicates were merged for further analysis. The contact maps with different resolutions were generated and balanced with an iterative correction and eigenvector method by Cooler (version 0.9.1) software. The “.hic” files were generated and normalized with the Knight-Ruiz (KR) balancing method by Juicer tools (version 1.13.02). The visualization of micro-C interactions was performed through the plotgardener package (version 1.4.2).

The cooltools eigs-cis (version 0.7.1) module was used for the analyses of A/B compartments with the observed/expected contact maps at 100-kb resolution in both zebrafish and African killifish. The eigenvectors were calculated, and the E1 vector was used to evaluate the compartmentalization pattern. Compartment transition was defined depending on the changes of compartment patterns (0 dpa to 2 dpa: “A to B” compartment or “B to A” compartment in the same genomic bins). The saddle plots were visualized with the Cooltools saddle module. The E1 vectors were sorted and combined into 40 groups. The top 2.5% E1 values and the bottom 2.5% E1 values were not considered to avoid potential outliers. The average observed/expected contact frequency between different groups was calculated and visualized as saddle plots. The strength and statistical significance of compartmentalization were calculated with the saddle strength function according to cooltools documentation.

The TAD boundaries and relevant insulation scores were identified and calculated by the cooltools insulation module with “.cool” files at 50-kb resolution. The visualization of TAD boundaries in the contact heatmap and the calculation of average insulation score around the boundary sites were performed by custom scripts. The R package TADcompare (version 1.8.0) was used for boundary comparison. For the stripe calling, the stripes were identified with .cool files at 5-kb resolution using StripeCaller (version 0.1.0), and the significance level was set as 0.01.

### Loop calling and classification

The chromatin loops were identified using Mustache (version 1.0.1) on KR-normalized .hic files at 5-kb resolution. The *P* value threshold for the loop identification was set as 0.1 (default threshold). The differential loops were also detected with Mustache software and verified manually in zebrafish and African killifish. The loop length was calculated by the distance between two loop anchors in the genome. The Pileup analyses of chromatin loops were performed by the coolpuppy (version 1.1.0) package. The significance of loop strength between 0 dpa and 2 dpa was calculated following the code from a previous study (*56*). The Homer (version 4.11) software was used for the motif enrichment analysis of loop anchors.

For loop type classification, the anchors of loops were assigned to active enhancers, active promoters, silencers, or others based on ATAC-seq data and H3K27ac ChIP-seq data. Anchors localized around ±1 kb of the transcription start site (TSS) and overlapped with open chromatin regions or H3K27ac peaks were classified as active promoters. Anchors overlapping H3K27ac peaks without active promoter annotation were defined as active enhancers. The genomic coordinates of regeneration silencer elements (TRSEs) in zebrafish were downloaded from a previous study (*40*). Anchors overlapped with TRSE regions but lacked active promoter or active enhancer annotation were classified as silencers. The remaining anchors were assigned to the “others” category.

### Chromosome conformation capture

The dissociated zebrafish caudal fin cells were fixed with 2% methanol-free formaldehyde (Pierce, 28906) solution for 10 min at RT and then quenched with glycine solution (final concentration: 0.125 M). Subsequently, these cells were resuspended in lysis buffer (50 mM tris-HCl, 0.5% NP-40, 1% Triton X-100, 150 mM NaCl, 5 mM EDTA, and 1× PIC) and chilled on ice for 20 min, followed by washing and resuspension in 1.2× NEBuffer DpnII (NEB, R0543M). The chromatin was released by adding SDS (final concentration: 0.3%) and incubated at 37°C for 1 hour. Then, Triton X-100 (final concentration: 2.5%) was added and incubated for 1 hour at 37°C. Subsequently, 200 U of DpnII (NEB, R0543M) was added to digest the cross-linked chromatin at 37°C overnight. Furthermore, the digested chromatin was diluted in 1× T4 ligation buffer (NEB, B0202S) and incubated with 4000 U of T4 ligase (NEB, M0202L) at 16°C overnight. After digesting with proteinase K (Thermo Fisher Scientific, EO0491) at 65°C and RNase A treatment (Vazyme, DE111-01) at 37°C, the ligation products were purified and detected by PCR with the following primers: 3C-zebrafish-Aldh1a2-F: TCACAGACAGATGGTGTCGCTCACAC; 3C-zebrafish-Aldh1a2-R: CAGGAAGGATTCCAAAGTTGTAATCGCAC; 3C-zebrafish-Mki67-F: GTTTGCTAAACAACTCGTCTCCCGACAG; 3C-zebrafish-Mki67-R: GCTGTCGTTATGCCTTCAATCTAGCACATG.

### Bulk RNA-seq

For zebrafish RNA-seq, we collected homeostatic tissues and regenerated tissues at 0 dpa and 2 dpa. Three biological replicates were prepared for each group. Six zebrafish were used for each biological replicate. The fresh tissues were washed with ice-cold DPBS and snap-frozen using liquid nitrogen. The tissues were homogenized in 1 ml of TRIzol reagent (Invitrogen, 15596026CN), and the subsequent total RNA extraction procedures were carried out according to the manufacturer’s protocol. The RNA-seq libraries were prepared using the NEBNext Ultra RNA library Prep Kit for Illumina (NEB, E7530L) and sequenced on the Illumina NovaSeq 6000 platform with 150-bp paired-end reads.

For African killifish and rabbit bulk RNA-seq, the raw data were obtained from previous studies (BioProject PRJNA559885 and PRJNA1055264) and realigned to the reference genome, respectively. Zebrafish, African killifish, and rabbit reference genomes and annotation files were the same as described above.

The bulk RNA-seq analyses were performed as previously described (*25*). Briefly, the alignment of raw reads was conducted using STAR (version 2.7.10a). The EdgeR (version 3.40.1) package was used for the analysis of differentially expressed genes (DEGs). Genes with the absolute value of log_2_(fold change) > 0.75 and FDR < 0.05 were considered as significant DEGs in this study. The GO term and KEGG enrichment analysis was carried out with the R package ClusterProfiler (version 4.6.0). The gene IDs of African killifish were converted to the corresponding zebrafish gene IDs according to the one-to-one orthologous gene list downloaded from the Ensembl Biomart database. The GO and KEGG terms with FDR < 0.05 were considered statistically enriched terms.

### ATAC-seq and ChIP-seq

For zebrafish and African killifish ATAC-seq experiments during fin regeneration, two replicates were generated in zebrafish, and three replicates were involved in African killifish, with five fish were used for each replicate at 0 dpa and 2 dpa. A total of 150,000 dissociated cells were harvested for each ATAC-seq sample. These cells were used for ATAC-seq with the Hyperactive ATAC-seq Library Prep Kit for Illumina (Vazyme, TD711-01) following the manufacturer’s protocol. For the ChIP-seq, the zebrafish and African killifish data were downloaded from NCBI BioProject PRJNA559885. Bowtie2 (version 2.4.5) was used to map the raw reads into the reference genome. The peaks of different ATAC-seq and ChIP-seq samples were identified by MACS2 (version 2.2.7.1). The R package DiffBind (version 3.8.3) was used for the differential peak identification, and the ChIPseeker (version 1.34.1) package was used for peak annotation.

### Single-cell RNA-seq data processing

We downloaded and reanalyzed the published zebrafish scRNA-seq data under the accession number GSE137971 (*22*). The raw reads were aligned to the zebrafish reference genome with Cell Ranger (version 7.0). The downstream analyses followed with Seurat recommended pipelines (version 4.3.0). After filtering the low-quality cells (nFeature_RNA < 300 and nFeature_RNA > 5000 and the percent of mitochondrial genes > 10%), the cell clustering was performed with the first 20 principal components (PCs) and the top 2000 variable genes. The resolution of the FindClusters function was set as 1. Different samples were integrated with the FindIntegrationAnchors function in Seurat. The Uniform Manifold Approximation and Projection plot was used to visualize the cell clustering results. The visualizations of gene expression were performed by the functions provided by the Seurat package.

### Single-cell ATAC-seq analysis

The scATAC-seq matrices of zebrafish caudal fin regeneration were downloaded from the NCBI GEO database under the accession number GSE261907 (*37*). The RNA and ATAC matrices were converted to Seurat objects using the Seurat and Signac packages, respectively (*57*, *58*). Low-quality cells were filtered before downstream analysis based on the following criteria: nFeature_RNA < 100, nFeature_RNA > 4000, percent.mt > 15%, nCount_ATAC > 200,000, nCount_ATAC < 200, and nucleosome_signal > 2, TSS.enrichment < 2. The scATAC-seq data were analyzed following the recommended Signac pipeline. Specifically, the peaks of scATAC-seq were recalled using the CallPeak function with MACS2. Then, the RunTFIDF, FindTopFeatures, and RunSVD functions were used for matrix normalization, finding top features, and linear dimensionality reduction. The joint neighbor graph of both scRNA-seq data and scATAC-seq data was constructed using the FindMultiModalNeighbors function with the first 50 PCs and 2 to 40 LSI components. The annotation of different cell types was performed on the basis of the marker genes described in a previous study (*37*). To assign RREs to specific cell types in scATAC-seq data, the marker peaks of different cell types were identified with the FindAllMarkers function in Seurat. The GenomicRanges (Version 1.50.2) package was used to intersect RREs with marker peaks based on their genomic coordinates.

### Genome synteny analysis

The synteny blocks between zebrafish and African killifish were identified by MCScanX (version 1.0.0) and visualized with Circos (version 0.69-9) software.

### Identification of conserved genes in A/B compartments

The one-to-one orthologous gene list between zebrafish and killifish was downloaded from the BioMart database of Ensembl 104. On the basis of the gene annotation of A/B compartment, we calculated the orthologous genes located in A and B compartment for zebrafish and African killifish. Then, we examined the number and proportion of shared orthologous genes located in the same type of compartment (A or B).

### Loop anchor deletion mediated by CRISPR-Cas9 genome editing system

The CRISPR-Cas9 system was adopted to knock out targeted loop anchors in African killifish genome. For RREs located in each loop anchor, two single-guide RNAs (sgRNAs) were designed using the CHOPCHOP website tool (https://chopchop.cbu.uib.no/) and subsequently synthesized and purified by GenScript Biotech (Nanjing, China). Then, the two sgRNAs were mixed with purified Cas9 nuclease (IDT, 1081059) and coinjected into one-cell–stage African killifish embryos according to a previously described protocol (*59*).

The sequences of sgRNAs for different loop anchors were as follows: K-RRE^il11a^-gRNA1 (ATTCAAGATAAGACACCCTG), K-RRE^il11a^-gRNA2 (ATCATAATGTTGGACGTCAG), K-RRE^fn1^-gRNA1 (GGATGCCTATCGAGTTCAGC), and K-RRE^fn1^-gRNA1 (GTTCGGCAGGCTTATCAAGG).

Two independent PCR amplifications were performed to genotype mutants with targeted deletion, followed by sequencing confirmation. The primers used for genotyping were as follows: RRE^fn1^-forward primer (ATAGTCCCGGGTACTCAAATTTAATGGTC), RRE^fn1^-reverse primer 1 (AACTTGAAGAGCTCTGCTTTGCC), RRE^fn1^-reverse primer 2 (AGCCAACAAGACCACATCGTCTG), RRE^il11a^-forward primer (ATGCTCACCTATTGTTCACATCTGG), RRE^il11a^-reverse primer 1 (GTCTCGCGTTGTCATGAATGC), and RRE^il11a^-reverse primer 2 (TAGGCGGTGAAACTCTAGGATTAG).

### Qualification of the regeneration of killifish caudal fin

Images of caudal fins were collected by Leica THUNDER Imaging Systems. Half of the caudal fin was removed in all animals. The length of the homeostatic caudal fins and regenerated caudal fins was measured using ImageJ for quantification. Normalized length of regenerated tissues was calculated, and the Student’s *t* test was performed to assess the statistical significance.

### Quantitative real-time PCR

The cDNA of knockout transgenic killifish lines was prepared using the HiScript III first-strand cDNA Synthesis Kit (Vazyme, R312). Quantitative real-time PCR was performed using Taq Pro Universal SYBR quantitative PCR (qPCR) Master Mix (Vazyme, Q712) according to the manufacturer’s instructions. The expression levels of the housekeeping gene *Gapdhs* were used for normalization. The qPCR primer sequences were as follows: K-il11a-qPCR-forward primer (AGCTCTCCAATCAGACCAAGAATC), K-il11a-qPCR-reverse primer (ACTCAAAGTGATGCTCGTACAGC), K-fn1-qPCR-forward primer (ATCACGCCTCCTCAGTCTCTC), K-fn1-qPCR-reverse primer (TCGCCCACATTGTAGTACCTCTG), K-umps-qPCR-forward primer (ACCTAAGGGTTCTGGTCTCCC), K-umps-qPCR-reverse primer (TGTCCTCAATGATCAGGCACG), K-gapdhs-qPCR-forward primer (CTTCCAGTGTATGAAGCCTGCTG), and K-gapdhs-qPCR-reverse primer (CACCCTGGATGTGAGACGAGG).

### African killifish transgenic reporter assays

The Tol2 transposon system was used to generate African killifish transgenic reporter lines. The sequence of *RRE^fn1^* and *Z-IEN2* was amplified from the African killifish and zebrafish genomes, respectively. Enhancers were cloned into the *pDest-Tol2-miniP-GFP-polyA-CV* reporter vector. Plasmid DNA used for injection was purified with the EndoFree Plasmid Maxi Kit (QIAGEN, 12362). Injections were done according to the previously published protocol (*59*). The images of transgenic reporter lines were acquired with Leica THUNDER Imaging Systems. The primer sequences used for RRE amplification were as follows: RRE^fn1^-forward primer (AAGGGGGACAACTCCAGATTGCTG), RRE^fn1^-reverse primer (TGAGGACCCCCTTGATAAGCCTGC), Z-IEN2-forward primer (CAATACCGTTTCAGTTAGTTCTAGAT), and Z-IEN2-reverse primer (CAGGACACTGTTATACAGCCTA).
