# Supplementary material for: Chromatin interaction landscape of epimorphic regeneration in teleost fish
Source: Sci Adv. 2026 Oct 9;12(41):eaeh2087. doi: 10.1126/sciadv.aeh2087 (PMC13651840; doi:10.1126/sciadv.aeh2087)
Supplement: Supplementary file 1 [file sciadv.aeh2087_sm.pdf]

Science Advances
AAAS

Supplementary Materials for

Chromatin interaction landscape of epimorphic regeneration in teleost fish

Xiaohui Jia *et al.*

Corresponding author: Wei Wang, wangwei@nibs.ac.cn

Sci. Adv. **12**, eaeh2087 (2026)
DOI: 10.1126/sciadv.aeh2087

The PDF file includes:

Figs. S1 to S6
Legends for tables S1 to S4

Other Supplementary Material for this manuscript includes the following:

Tables S1 to S4

Figure S1

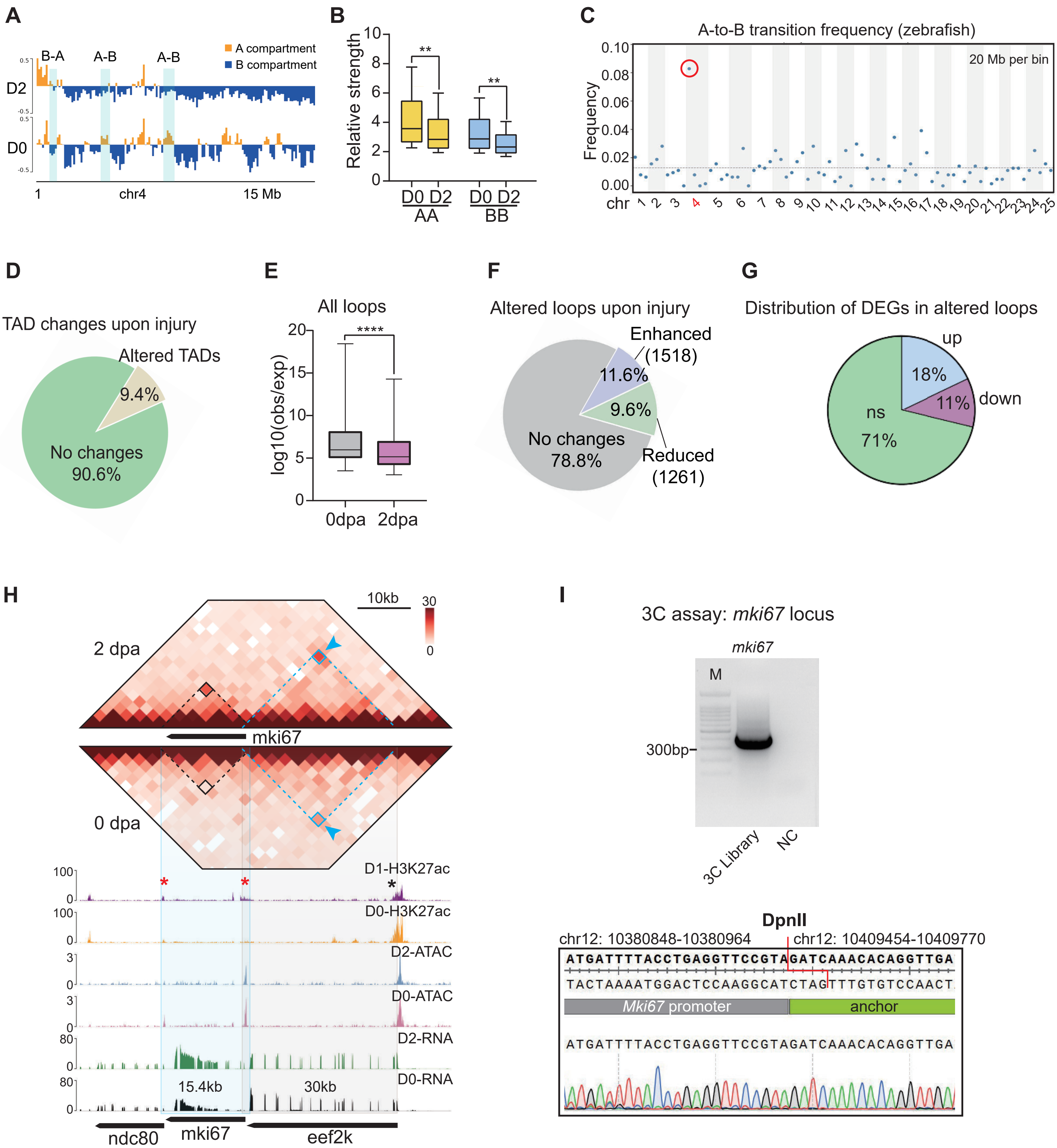

A
B-A
A-B
A-B
A compartment
B compartment
D2
D0
1
chr4
15 Mb
B
Relative strength
D0 D2
AA
D0 D2
BB
C
A-to-B transition frequency (zebrafish)
Frequency
20 Mb per bin
chr
D
TAD changes upon injury
Altered TADs
9.4%
No changes
90.6%
E
All loops
log10(obs/exp)
0dpa
2dpa
F
Altered loops upon injury
Enhanced
(1518)
11.6%
9.6%
No changes
78.8%
Reduced
(1261)
G
Distribution of DEGs in altered loops
up
18%
11%
down
ns
71%
H
2 dpa
0 dpa
10kb
mki67
D1-H3K27ac
D0-H3K27ac
D2-ATAC
D0-ATAC
D2-RNA
D0-RNA
15.4kb
30kb
ndc80
mki67
eef2k
I
3C assay: mki67 locus
mki67
M
300bp
3C Library
NC
DpnII
chr12: 10380848-10380964
chr12: 10409454-10409770
ATGATTTTACCTGAGGTTCCGTAGATCAAACACAGGTTGA
TACTAAAATGGACTCCAAGGCATCTAGTTTGTGTCCAACT
Mki67 promoter
anchor
ATGATTTTACCTGAGGTTCCGTAGATCAAACACAGGTTGA

Fig S1. Reorganization of chromatin architecture upon zebrafish caudal fin amputation. (**A**) A representative region on the chromosome 4 showing B-to-A and A-to-B compartment transitions. (**B**) Quantification of AA and BB compartment strength at 0 dpa versus 2 dpa in zebrafish. (***P* value < 0.01, paired Student's *t*-test). **(C)** Distribution of A-to-B compartment transition frequency across chromosomes following injury. Each bin represents 20-Mb region. The dashed line represents the random frequency. The first bin of the chromosome 4 displayed the highest frequency of A-to-B compartment transitions. (**D**) The proportion of TAD changes upon zebrafish caudal fin amputation. Most TADs (90.6%) remained stable. (**E**) Quantification of piled-up all-loops signal in zebrafish (*****P* value < 0.0001, paired two-tailed *t*-test). (**F**) The proportion of injury-altered chromatin loop interactions upon zebrafish caudal fin injury. (**G**) The proportion of differentially expressed genes associated with altered loops in zebrafish. (**H**) An enhanced promoter-promoter interaction (arrowhead) was detected at the *mki67* locus during zebrafish fin regeneration. Gene expression and active enhancers were visualized through the RNA-seq peaks and H3K27ac peaks, respectively. Red star indicates up-regulated H3K27ac peaks, while black star indicates down-regulated H3K27ac peaks. (**I**) Validation of the upstream promoter-promoter interaction highlighted in panel H (blue) using 3C assays. The top panel is the agarose gel indicating the ligated product of *mki67* promoter and the other loop anchor. The bottom panel shows the sequencing result of the ligated product.

Figure S2

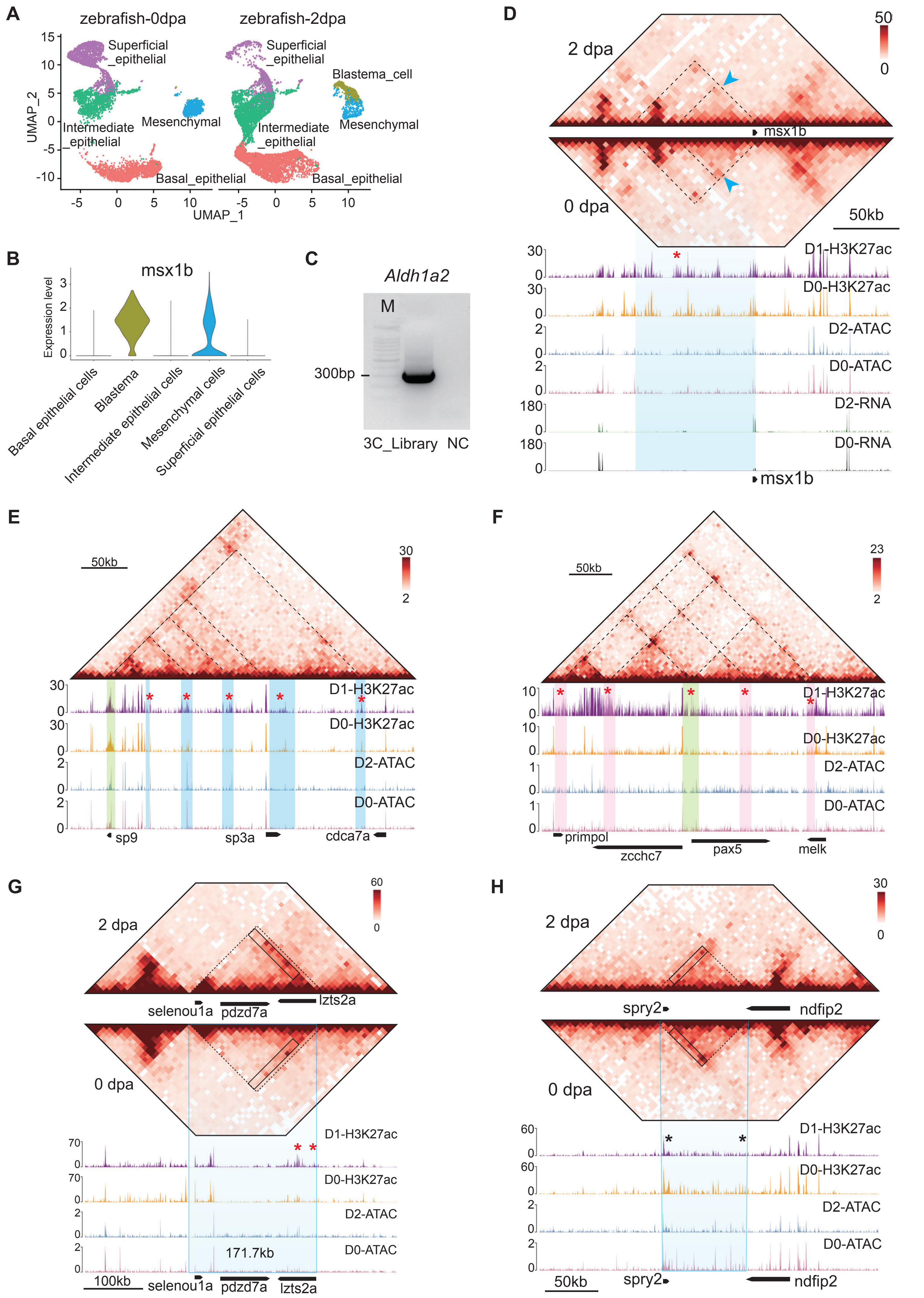
A
zebrafish-0dpa
zebrafish-2dpa
Superficial_epithelial
Intermediate_epithelial
Mesenchymal
Basal_epithelial
Blastema_cell
UMAP_1
UMAP_2
B
msx1b
Expression level
Basal epithelial cells
Blastema
Intermediate epithelial cells
Mesenchymal cells
Superficial epithelial cells
C
Aldh1a2
M
300bp
3C_Library
NC
D
2 dpa
0 dpa
msx1b
50kb
D1-H3K27ac
D0-H3K27ac
D2-ATAC
D0-ATAC
D2-RNA
D0-RNA
E
50kb
sp9
sp3a
cdca7a
F
50kb
primpol
zcchc7
pax5
melk
G
2 dpa
0 dpa
selenou1a
pdzd7a
lzts2a
171.7kb
100kb
H
spry2
ndfip2
50kb

Fig S2. Chromatin architectural stripes detected in zebrafish. (**A**) The UMAP plot shows the major cell populations identified at 0 dpa and 2 dpa during zebrafish caudal fin regeneration. The fin blastema cells were specific to the 2-dpa sample. (**B**) Violin plot showing the expression of *msx1b* in different cell populations. (**C**) The agarose gel showing the ligated product of *aldh1a2* promoter and the other loop anchor. (**D**) A reduced promoter-enhancer interaction (arrowhead) was detected at the *msx1b* locus during zebrafish fin regeneration. Gene expression and active enhancers were visualized through the RNA-seq peaks and H3K27ac peaks, respectively. *up-regulated peaks. (**E**) The architectural stripe detected at the *sp9* locus. The promoter of *sp9* (green) interacted with multiple enhancers with increased H3K27ac peaks (blue) at 2 dpa. *up-regulated peaks. (**F**) The architectural stripe detected at the *primpol* locus at 2 dpa. Nested enhancer-enhancer interactions were formed. Active enhancers were visualized through the H3K27ac peaks. *up-regulated peaks. (**G**) Formation of chromatin stripe architecture at *lzts2a* locus upon amputation. *up-regulated peaks. (**H**) Disruption of chromatin stripe architecture at *spry2* locus upon amputation. *down-regulated peaks.

Figure S3

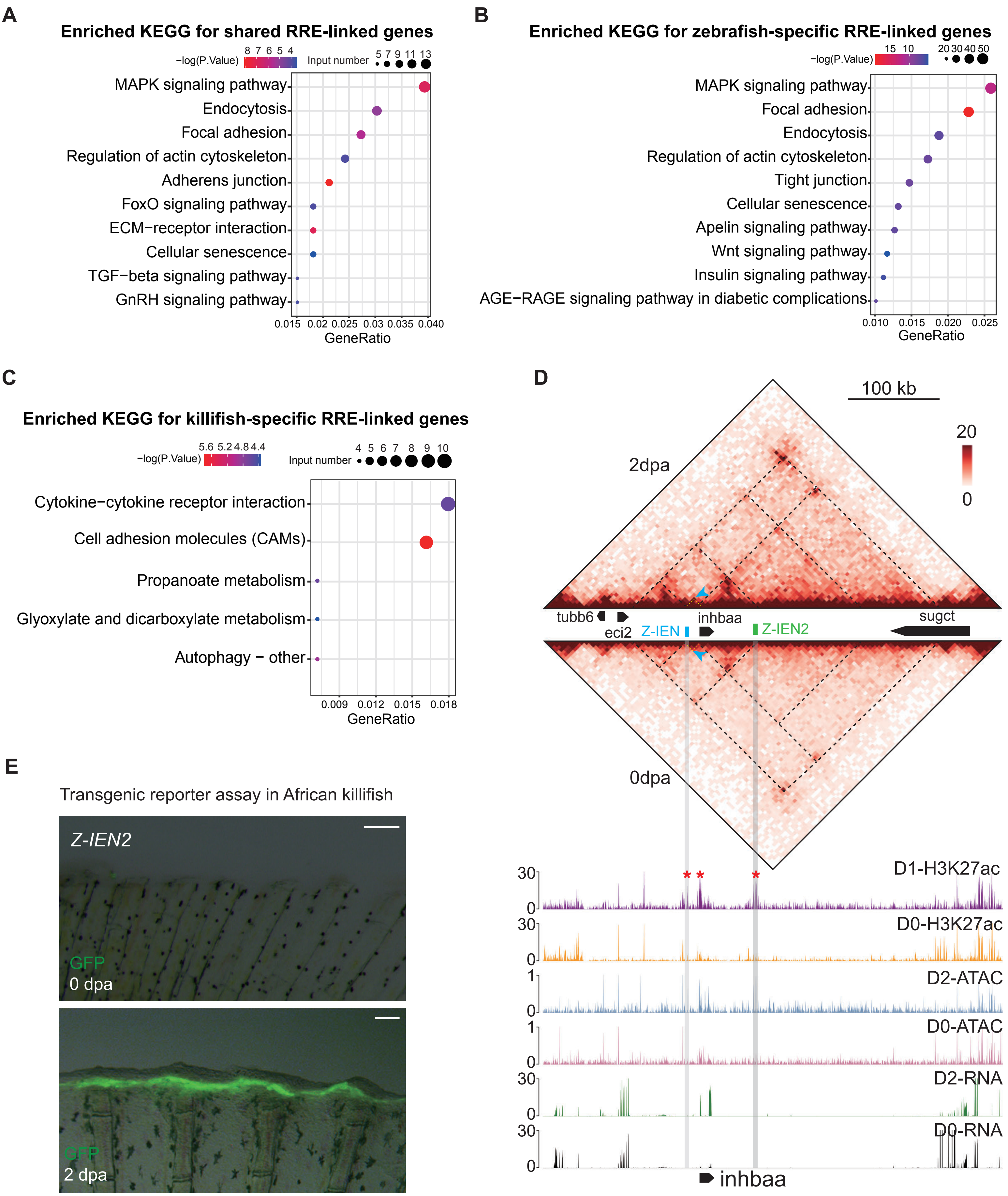

A
Enriched KEGG for shared RRE-linked genes
−log(P.Value)
8 7 6 5 4
Input number
5 7 9 11 13
MAPK signaling pathway
Endocytosis
Focal adhesion
Regulation of actin cytoskeleton
Adherens junction
FoxO signaling pathway
ECM−receptor interaction
Cellular senescence
TGF−beta signaling pathway
GnRH signaling pathway
0.015 0.02 0.025 0.03 0.035 0.040
GeneRatio
B
Enriched KEGG for zebrafish-specific RRE-linked genes
−log(P.Value)
15 10
20 30 40 50
MAPK signaling pathway
Focal adhesion
Endocytosis
Regulation of actin cytoskeleton
Tight junction
Cellular senescence
Apelin signaling pathway
Wnt signaling pathway
Insulin signaling pathway
AGE−RAGE signaling pathway in diabetic complications
0.010 0.015 0.020 0.025
GeneRatio
C
Enriched KEGG for killifish-specific RRE-linked genes
−log(P.Value)
5.6 5.2 4.8 4.4
Input number
4 5 6 7 8 9 10
Cytokine−cytokine receptor interaction
Cell adhesion molecules (CAMs)
Propanoate metabolism
Glyoxylate and dicarboxylate metabolism
Autophagy − other
0.009 0.012 0.015 0.018
GeneRatio
D
100 kb
20
0
2dpa
tubb6
eci2
Z-IEN
inhbaa
Z-IEN2
sugct
0dpa
30
0
D1-H3K27ac
D0-H3K27ac
1
D2-ATAC
D0-ATAC
D2-RNA
D0-RNA
inhbaa
E
Transgenic reporter assay in African killifish
Z-IEN2
GFP
0 dpa
GFP
2 dpa

Fig S3. Identification of regeneration-responsive enhancers. (**A**) Representative KEGG terms enriched for the shared *RRE*-linked genes between zebrafish and African killifish. (**B**) Representative KEGG terms enriched for zebrafish-specific *RRE*-linked genes. (**C**) Representative KEGG terms enriched for killifish-specific *RRE*-linked genes. (**D**) Complex chromatin loop interactions detected at the *inhbaa* locus. *Z-IEN* is a previously characterized RRE. *Z-IEN2* is a new RRE detected in this study and interacted with the *inhbaa* promoter. *up-regulated peaks. (**E**) *Z-IEN2* was sufficient to drive GFP expression at the wounding site at 2 dpa during fin regeneration. Scale bar, 250 μm.

Figure S4

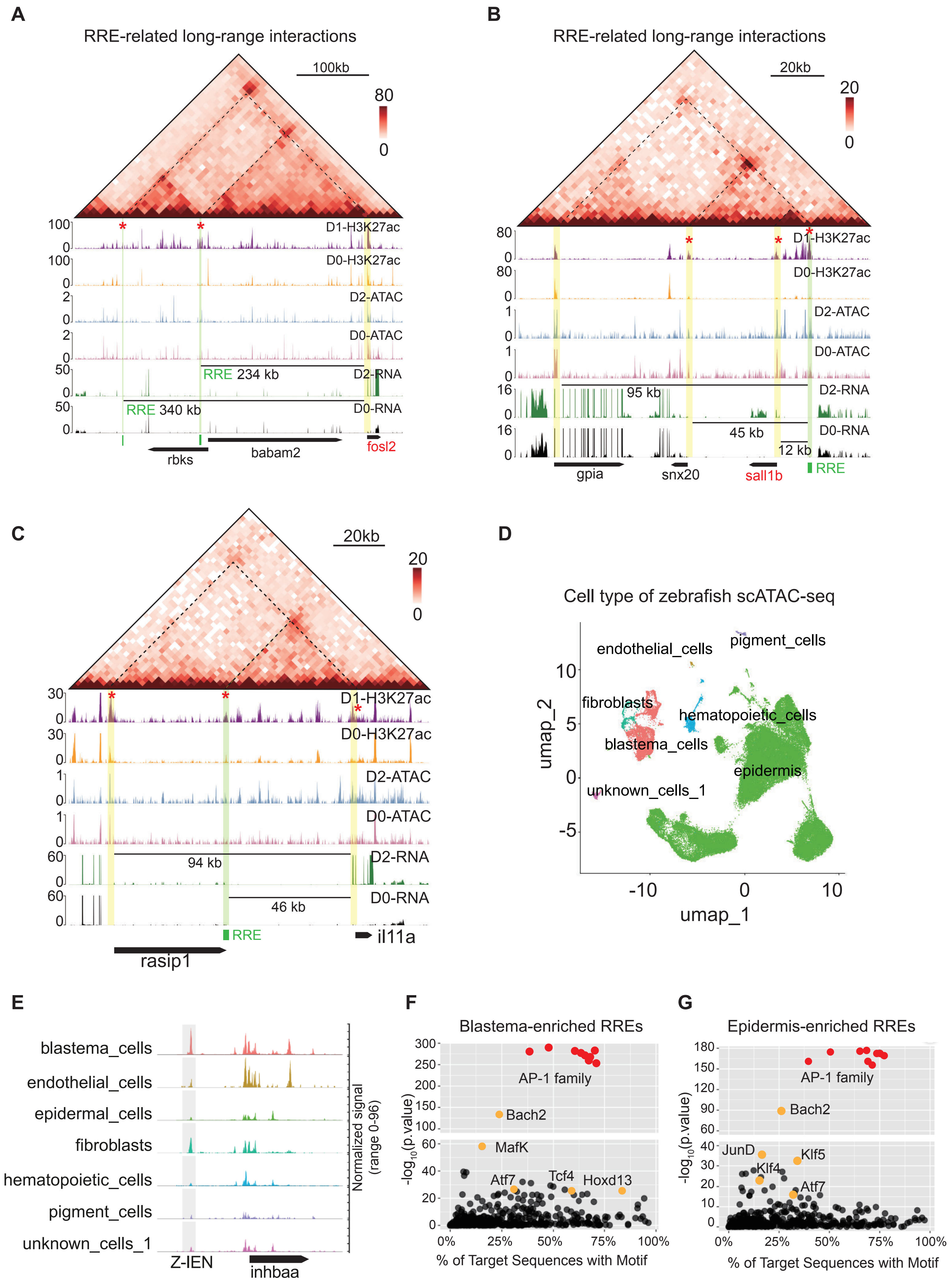

A
RRE-related long-range interactions
100kb
80
0
D1-H3K27ac
D0-H3K27ac
D2-ATAC
D0-ATAC
D2-RNA
D0-RNA
RRE 234 kb
RRE 340 kb
rbks
babam2
fosl2
B
RRE-related long-range interactions
20kb
20
0
D1-H3K27ac
D0-H3K27ac
D2-ATAC
D0-ATAC
D2-RNA
D0-RNA
95 kb
45 kb
12 kb
gpia
snx20
sall1b
RRE
C
20kb
20
0
D1-H3K27ac
D0-H3K27ac
D2-ATAC
D0-ATAC
D2-RNA
D0-RNA
94 kb
46 kb
RRE
il11a
rasip1
D
Cell type of zebrafish scATAC-seq
endothelial_cells
pigment_cells
fibroblasts
hematopoietic_cells
blastema_cells
epidermis
unknown_cells_1
umap_2
umap_1
E
blastema_cells
endothelial_cells
epidermal_cells
fibroblasts
hematopoietic_cells
pigment_cells
unknown_cells_1
Normalized signal (range 0-96)
Z-IEN
inhbaa
F
Blastema-enriched RREs
AP-1 family
Bach2
MafK
Atf7
Tcf4
Hoxd13
-log10(p.value)
% of Target Sequences with Motif
G
Epidermis-enriched RREs
AP-1 family
Bach2
JunD
Klf5
Klf4
Atf7
-log10(p.value)
% of Target Sequences with Motif

Fig S4. RRE-linked chromatin loop interactions during zebrafish regeneration. (**A**) Long-range interactions detected between the *fosl2* promoter and two distal RREs. *up-regulated peaks. (**B**) Long-range interactions detected at the *sall1b* locus. A RRE located 12-kb upstream of *sall1b* interacted with the promoters of *gpia*, *snx20*, and *sall1b*. *up-regulated peaks. (**C**) Long-range interactions detected at the *il11a* locus. A RRE located 46-kb upstream of *il11a* promoter interacted with the *il11a* promoter. The *rasip1* promoter with increased H3K27ac peak displayed interactions with the *il11a* promoter. *up-regulated peaks. (**D**) Uniform manifold approximation and projection (UMAP) plot showing the major cell population in zebrafish scATAC-seq dataset. (**E**) Aggregated and normalized scATAC-seq signals of *Z-IEN* in different cell types. (**F**) Motif enrichment analysis for blastema-enriched RREs. (**G**) Motif enrichment analysis for epidermis-enriched RREs.

Figure S5

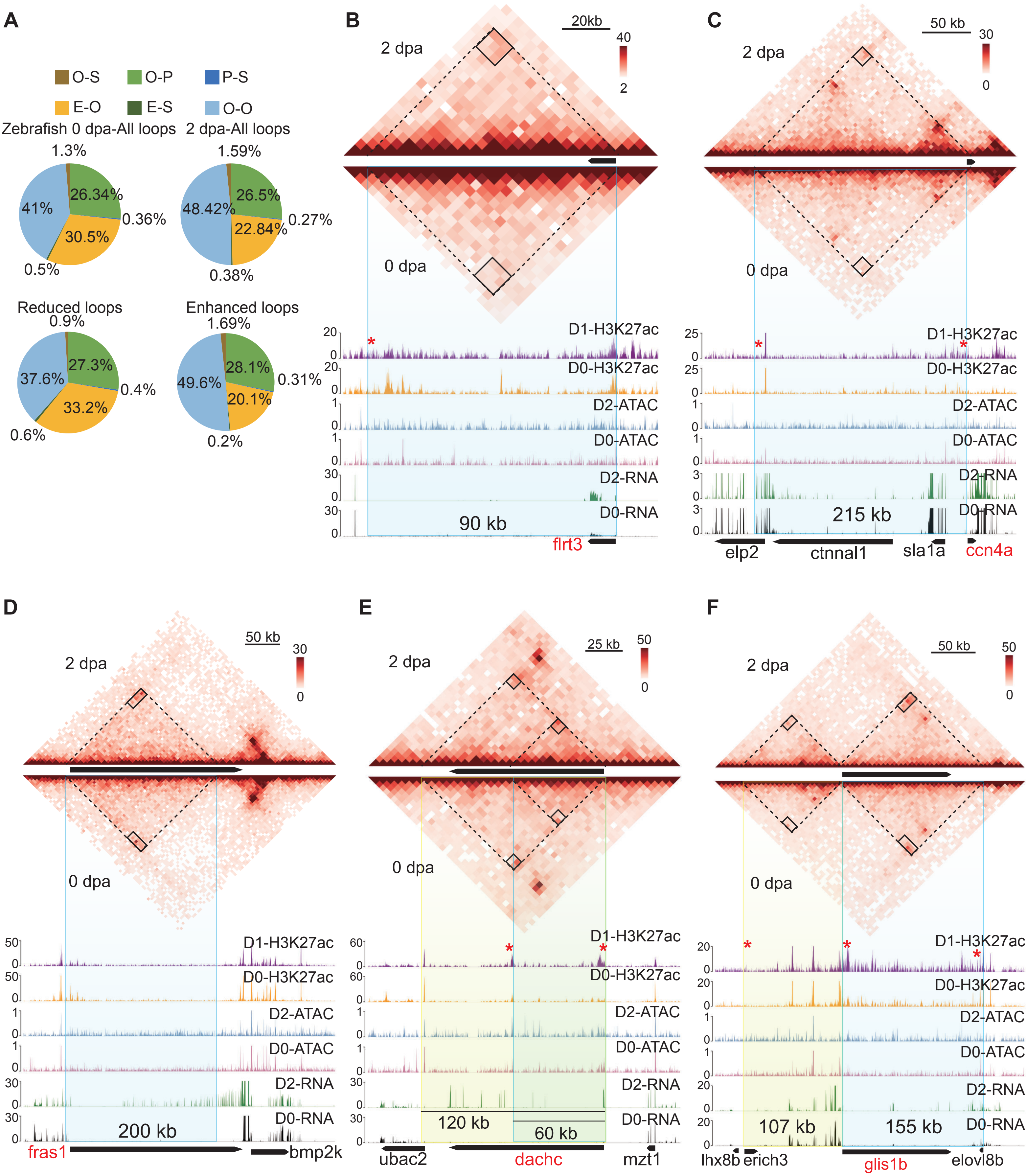
A
O-S
O-P
P-S
E-O
E-S
O-O
Zebrafish 0 dpa-All loops
2 dpa-All loops
1.3%
26.34%
41%
0.36%
30.5%
0.5%
1.59%
26.5%
48.42%
0.27%
22.84%
0.38%
Reduced loops
0.9%
27.3%
37.6%
0.4%
33.2%
0.6%
Enhanced loops
1.69%
28.1%
49.6%
0.31%
20.1%
0.2%
B
2 dpa
20kb
40
2
0 dpa
D1-H3K27ac
D0-H3K27ac
D2-ATAC
D0-ATAC
D2-RNA
D0-RNA
90 kb
flrt3
C
2 dpa
50 kb
30
0
0 dpa
215 kb
elp2
ctnnal1
sla1a
ccn4a
D
2 dpa
50 kb
0 dpa
200 kb
fras1
bmp2k
E
2 dpa
25 kb
50
0 dpa
120 kb
60 kb
ubac2
dachc
mzt1
F
2 dpa
50 kb
0 dpa
107 kb
155 kb
lhx8b
erich3
glis1b
elovl8b

Fig S5. RRGs associated with Long-range chromatin interactions during zebrafish regeneration. (**A**) The proportion of different types of loop interaction among 'Others' type. P-S: promoter-silencer interaction, E-S: enhancer-silencer interaction. 'O' refers to the interactions involving non-promoter/enhancer/silencer-related elements. (**B**) Chromatin architecture at the *flrt3* locus. An enhanced long-range interaction was detected between the *flrt3* promoter and a region 90 kb downstream at 2 dpa. *up-regulated peaks. (**C**) Chromatin architecture at the *ccn4a* locus. An enhanced long-range interaction was identified between the *ccn4a* promoter and a region 215 kb upstream at 2 dpa. *up-regulated peaks. (**D**) Chromatin architecture at the *fras1* locus. An enhanced long-range interaction was identified between the *fras1* promoter and a region 200 kb downstream at 2 dpa. (**E**) Chromatin architecture at the *dachc* locus. An enhanced long-range interaction was identified between the *dachc* promoter and two downstream genomic regions (60 kb and 120 kb) at 2 dpa. *up-regulated peaks. (**F**) Chromatin architecture at the *glis1b* locus. An enhanced long-range interaction was identified between the *glis1b* promoter and two distal regions (155 kb downstream and 107 kb upstream) at 2 dpa. *up-regulated peaks.

Figure S6

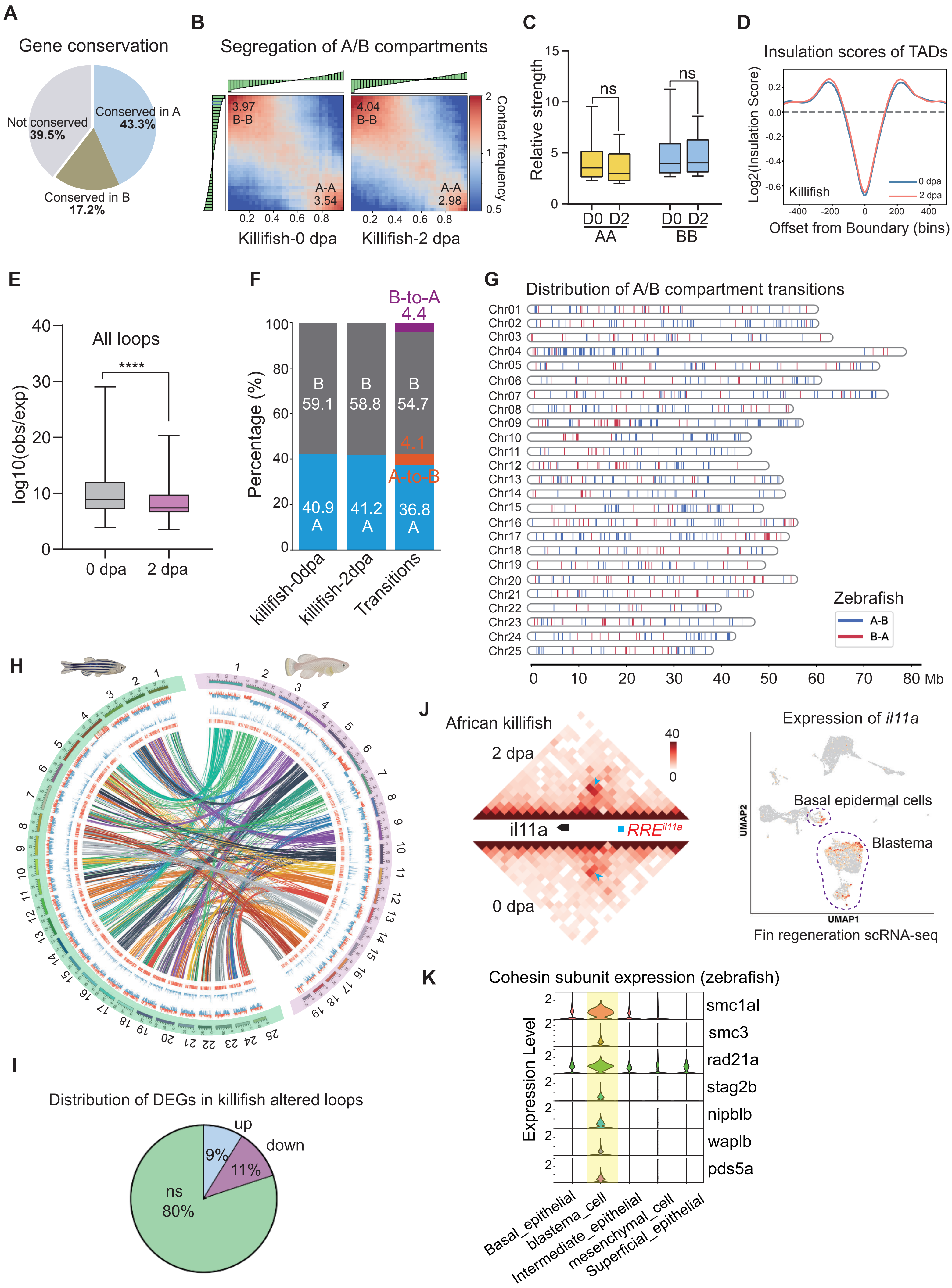
A
Gene conservation
Conserved in A 43.3%
Not conserved 39.5%
Conserved in B 17.2%
B
Segregation of A/B compartments
3.97 B-B
A-A 3.54
4.04 B-B
A-A 2.98
Contact frequency
Killifish-0 dpa
Killifish-2 dpa
C
Relative strength
ns
ns
D0 D2 AA
D0 D2 BB
D
Insulation scores of TADs
Log2(Insulation Score)
Killifish
0 dpa
2 dpa
Offset from Boundary (bins)
E
All loops
log10(obs/exp)
****
0 dpa
2 dpa
F
Percentage (%)
B-to-A 4.4
B 59.1
B 58.8
B 54.7
4.1
A-to-B
40.9 A
41.2 A
36.8 A
killifish-0dpa
killifish-2dpa
Transitions
G
Distribution of A/B compartment transitions
Zebrafish
A-B
B-A
Mb
H
J
African killifish
2 dpa
0 dpa
il11a
RRE il11a
Expression of il11a
Basal epidermal cells
Blastema
UMAP1
UMAP2
Fin regeneration scRNA-seq
I
Distribution of DEGs in killifish altered loops
up 9%
down 11%
ns 80%
K
Cohesin subunit expression (zebrafish)
Expression Level
smc1al
smc3
rad21a
stag2b
nipblb
waplb
pds5a
Basal_epithelial
blastema_cell
Intermediate_epithelial
mesenchymal_cell
Superficial_epithelial

Fig S6. Comparison of chromatin architecture in regenerative species. (**A**) The percentage of one-to-one conserved orthologous genes located in the same type of A/B compartments between zebrafish and African killifish. (**B**) Saddle plots showing the genome-wide interaction at 0 and 2 dpa in African killifish. (**C**) Quantification of changes in the AA and BB compartment strength between 0 dpa and 2 dpa in African killifish. ns, not significant. The paired Student's *t*-test was performed. (**D**) Comparison of the average insulation scores around TAD boundaries between 0 dpa and 2 dpa in African killifish. (**E**) Quantification of piled-up all loops signal in African killifish (****P value < 0.0001, paired two-tailed *t*-test). (**F**) Bar plot showing the proportion of A/B compartments at 0 and 2 dpa and the compartment transitions (B-to-A and A-to-B) in African killifish. (**G**) The genomic distribution of compartment transitions (A-to-B and B-to-A) in zebrafish at 2 dpa. Red, B-to-A transition. Blue, A-to-B transition. All transitions were plotted on each chromosome. (**H**) The syntenic blocks between zebrafish and African killifish, highlighting the distinct chromosomal conservation at different genomic regions. Species icons were created in BioRender. Yang, J. (2026) https://BioRender.com/bqn7p2k. (**I**) The proportion of differentially expressed genes associated with altered loops in African killifish. (**J**) An increased chromatin interaction between *il11a* promoter and RRE^{il11a} was detected during African killifish fin regeneration. Right, UMAP showing *il11a* was expressed in the blastema and basal epidermal cell during African killifish fin regeneration. (**K**) Violin plots showing the expression of cohesion subunit genes including *smc1al*, *smc3*, *rad21a*, *stag2b*, *nipblb*, *waplb*, *pds5a* in zebrafish regenerating blastema.

Supplementary Tables

Supplementary table 1. Chromatin loop interactions identified during zebrafish fin regeneration.

Supplementary table 2. Regeneration-responsive genes and blastema genes identified during zebrafish regeneration.

Supplementary table 3. Chromatin architectural stripes detected in zebrafish.

Supplementary table 4. Chromatin loop interactions identified during African killifish fin regeneration.
